# Cardiotoxicity of small-molecule kinase inhibitors in cancer therapy

**DOI:** 10.1186/s40164-025-00660-5

**Published:** 2025-05-09

**Authors:** Shuangli Zhu, Kai Fu, Sijia Li, Chuan Yang, Can Pan, Xueping Wang, Fang Wang, Xiyong Yu, Kenneth Kin Wah To, Liwu Fu

**Affiliations:** 1 State Key Laboratory of Oncology in South China, Collaborative Innovation Center for Cancer Medicine, Guangdong Esophageal Cancer Institute, Guangdong Provincial Clinical Research Center for Cancer, Sun Yat-Sen University Cancer Center, Guangzhou, 510060 Guangdong People’s Republic of China; 2 Key Laboratory of Molecular Target & Clinical Pharmacology and the State Key Laboratory of Respiratory Disease, School of Pharmaceutical Sciences & The Fifth Affiliated Hospital, Guangdong, Guangzhou Medical University, Guangzhou, 511436 China; 3 School of Pharmacy, The Chinese University of Hong Kong, Hong Kong, 999077 China

**Keywords:** Cancer, Small-molecule kinase inhibitors, Cardiotoxicity

## Abstract

Cancer is one of the leading causes of death worldwide. Recent advances in precision oncology have enabled many specific cancer patient populations to respond well and achieve longer survival with small-molecule kinase inhibitors, which have become a new therapeutic strategy for tumors. Since 2001, the Food and Drug Administration has approved 108 and 63 new anticancer drugs for treating solid tumors and hematological malignancies, respectively, 89 of which belong to the large group of small-molecule kinase inhibitors (SMKIs). Compared to conventional chemotherapeutic agents such as cyclophosphamide, doxorubicin, and 5-FU, SMKIs offer better efficacy with fewer toxic side effects. Nevertheless, with the development of more novel SMKIs and their wider clinical application to a larger population of cancer patients, variable degrees of cardiotoxic adverse events have emerged for some SMKIs during cancer therapy. This review comprehensively summarizes the most updated progress in the cardiotoxicity of SMKIs in cancer therapy and discusses the new findings and mechanisms, which will provide emerging strategies for the prevention of cardiotoxicity caused by small molecule targeted drugs and the design of the next generation of low cardiotoxicity targeted drugs.

## Introduction

**Fig. 1:**
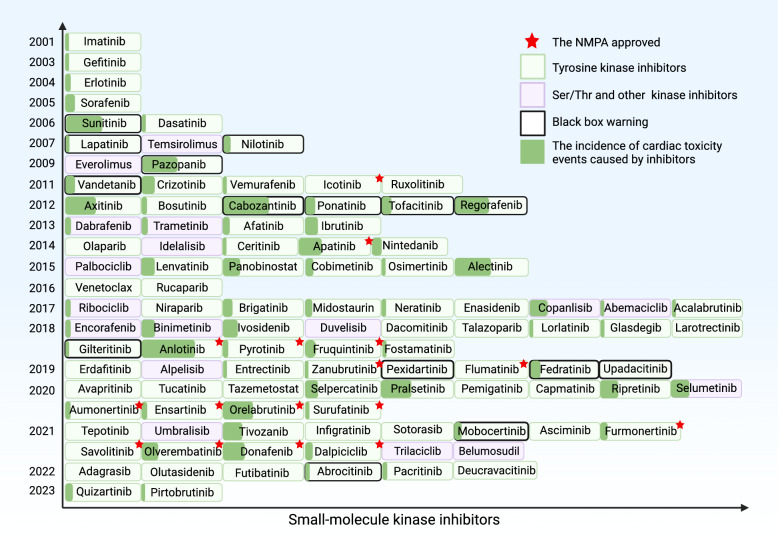
Timeline for the FDA and NMPA approval of small-molecule kinase inhibitors. The figure shows the small-molecule kinase inhibitors approved by the FDA and NMPA from 2001 to 2023. Except for the red star representing small-molecule inhibitors approved by NMPA, all others are FDA-approved. The green box shows tyrosine kinase inhibitors, while the pink box shows ser/thr kinase inhibitors, and the black box shows drugs with black box warnings. Green represents the incidence of cardiotoxicity events induced by small molecule inhibitors

Based on GLOBOCAN 2020 data, approximately 4.82 million and 2.37 million new cancer cases, and 3.21 million and 0.64 million cancer deaths were reported in China and the United States of America in 2022, respectively [1–3]. Traditional treatment strategies, including surgery, radiotherapy, chemotherapy, and immunotherapy, are still the mainstay of cancer therapy. However, tumor recurrence, drug resistance, distant metastasis, radiation resistance, and treatment-related serious adverse events are the main causes of failure of conventional cancer therapies. With the advances in medical science research in recent years, molecular targeted therapy and cancer immunotherapy have shown unprecedented success in the treatment of various cancer types [4]. Following the Food and Drug Administration (FDA) approval of the first-in-class tyrosine kinase inhibitor (TKI) imatinib for the treatment of chronic myeloid leukemia in 2001, there has been a growing interest in the development of targeted drugs for cancer treatment [5]. Chemotherapy agents, such as cyclophosphamide, doxorubicin, and 5-FU, primarily target various stages of tumor cell proliferation, effectively inhibiting or eradicating malignant cells. Unlike conventional chemotherapy, targeted drugs are capable of specifically recognizing tumor cells and effectively impeding cancer growth and proliferation. Cancer treatment with targeted drugs is more precise, and there is a lower chance of developing toxic side effects [6]. Consistently, the number and proportion of targeted drugs approved by the FDA have increased significantly (Fig. 1).

Targeted therapeutics are broadly divided into small molecules and large molecules. Small molecular drugs are chemicals with low molecular weight, which are primarily kinase inhibitors (receptor TKIs and non-receptor TKIs). On the other hand, large molecular drugs are usually made of proteins that are manufactured or extracted from living organisms. They encompass monoclonal antibodies and antibody-drug conjugates [7, 8]. Since 2001, the FDA has approved 108 new anticancer agents for solid tumors and 63 for hematologic malignancies. Among these newly approved anticancer drugs, 89 are small molecular drugs [9]. Small-molecule kinase inhibitors (SMKIs) are usually smaller than 500 Da in molecular weight, thus allowing their efficient penetration across cell membranes. Moreover, they are usually administered orally to facilitate better patient compliance, and they are also substantially less expensive than the large molecular biologic drugs [10].

**Fig. 2:**
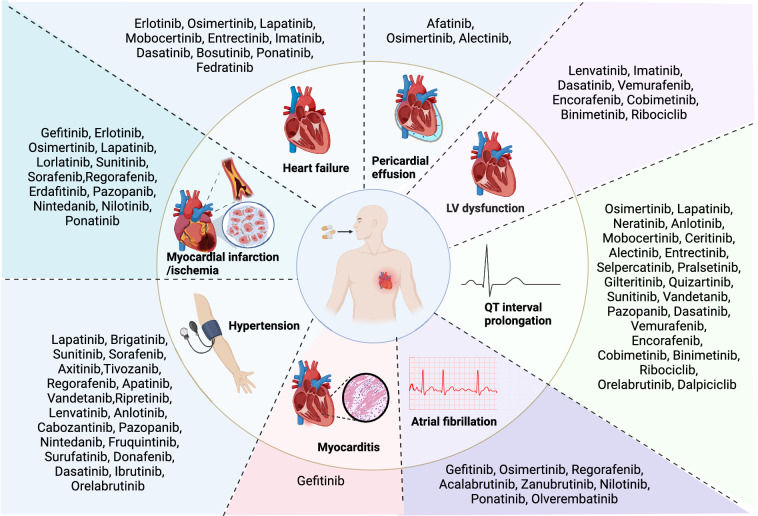
Cardiovascular toxicities of small-molecule kinase inhibitors. The figure shows the cardiotoxicity events induced by small molecule inhibitors, including myocarditis, atrial fibrillation, hypertension, QT interval prolongation, LV dysfunction, pericardial effusion, heart failure, and myocardial infarction or ischemia. We have identified small molecule inhibitors that induce corresponding cardiotoxicity

Cardiovascular toxicity, such as heart failure (HF), arrhythmias, and coronary artery disease, represent notable adverse effects of cancer therapy [11]. (Fig. 2) They are becoming more prominent with the longer duration of cancer treatment, which seriously affects the therapeutic effect and prognosis of cancer patients [12]. For example, TKIs and angiogenesis inhibitors are known to cause cardiotoxicity in cancer patients [13]. It has been estimated that approximately 62.92% of SMKIs may potentially mediate adverse cardiac events. Substantial research has been conducted to understand the etiology of cancer treatment-related cardiotoxic events. However, the specific mechanism(s) inducing cardiotoxicity by targeted anticancer drugs remain to be further explored.

This review will provide an updated summary of the cardiotoxicity caused by the clinically approved SMKIs. We will discuss the latest research on the mechanisms responsible for cardiotoxic effects in cancer patients and highlight novel strategies for their management.

## Receptor tyrosine kinase inhibitors

### Epidermal growth factor receptor (EGFR) inhibitors

Non-small cell lung cancer (NSCLC) is a histological subtype of lung cancer constituting more than 85% of all cases. Numerous EGFR mutations (e.g., exon 19 deletion or L858R mutation) are constitutively active and they drive “addictive” oncogenic signaling in cancer cells. EGFR TKIs are developed to target this “addictive” signaling and disrupt the downstream pathways to selectively kill cancer cells [14]. While EGFR TKIs are generally well tolerated, cardiotoxicities (including QT interval prolongation, left ventricular ejection fraction (LVEF) reduction, myocardial infarction (MI), atrial fibrillation (AF), and heart failure (HF)) have been reported for some TKIs [15] (Table 1).

**Table 1:** The cardiotoxicity of EGFR-TKIs

Generation	Name	Chemical structure	Targets	Approved for indications on	Cardiotoxicity	Corporation
EGFR						
First-	Gefitinib (Iressa)		EGFR	NSCLC 05/05/2003	MI, myocarditis, cardiac hypertrophy, AF, left bundle branch block	AstraZeneca
	Erlotinib (Tarceva)		EGFR	NSCLC (2004) Pancreatic cancer (2005)	Myocardial ischemic, MI, HF, venous thromboembolism	Roche/Astellas
	Icotinib (Conmana)		EGFR	NSCLC 06/07/2011	ND	BettaPharma
Second-	Afatinib (Gilotrif)		EGFR	NSCLC 07/2013	diastolic dysfunction, left ventricle enlargement, pericardial effusion	Boehringer Ingelheim
	Dacomitinib (Vizimpro)		EGFR	NSCLC 10/2018	ND	Pfizer
Third-	Osimertinib (Tagrisso)		EGFR	NSCLC 11/2015	Acute coronary syndrome, QT interval prolongation, MI, HF, mitral or tricuspid valve regurgitation, AF, pericardial effusion, decreased LVEF, supraven-tricular tachycardia	AstraZeneca
	Aumonertinib (Ameile)		EGFR	NSCLC 2020	QT interval prolongation	Hansoh
	Furmonertinib (Alflutinib)		EGFR	NSCLC 03/2021	QT interval prolongation, AF	Allist Pharmaceuticals
HER						
HER2	Lapatinib (Tykerb)		EGFR/HER2	Breast cancer 03/2007	Cardiomyopathy, hypotension, arrhythmia, ischemic heart disease, QT prolongation, decreased LVEF, HF	Novartis
	Neratinib (Nerlynx)		EGFR/HER2/HER4	Breast cancer 07/2017	Arrhythmia, ischemic heart disease, QT interval prolongation, decreased LVEF, HF	Puma Biotech
	Tucatinib (Tukysa)		HER2	HER2-positive breast cancer 04/2020	ND	Seagen
	Mobocertinib (Exkivity)		EGFR/HER2	NSCLC 09/2021	QT interval prolongation, HF	Takeda Pharmaceutical Company
	Pyrotinib		pan-HER	HER2-positive breast cancer 08/2018	QT interval prolongation	Hengrui Medicine

All data statistics from the FDA (https://www.accessdata.fda.gov)

EGFR: epidermal growth factor receptor; NSCLC: non-small cell lung cancer; MI: myocardial infarction; AF: arial fibrillation; HF: heart failure; HER2: human epidermal growth factor receptor 2; LVEF: left ventricular ejection fraction; ND: No data

#### The first-generation EGFR-TKIs

EGFR-TKIs are widely used to treat NSCLC patients. Erlotinib, gefitinib, and icotinib are the first-generation EGFR-TKIs that bind reversibly to EGFR and compete with ATP for binding to the TK domain.

##### Gefitinib

According to the analysis results of the FDA Adverse Event Reporting System (AERS) database, the incidence of cardiotoxicity induced by gefitinib is approximately 1.6% [16]. A series of case reports found that gefitinib could induce cardiotoxicity such as MI, left bundle branch block, AF, myocarditis, and venous thromboembolism [17–20]. 

Gefitinib was found to enhance the expression of phosphatase and tensin homolog (PTEN) in cardiomyocytes, while also increasing the expression of FoxO3a through the inhibition of the PI3K/AKT signaling pathway, ultimately leading to cardiotoxicity [21]. In a rat cardiotoxicity model, gefitinib induced cardiac hypertrophy by elevating the expression of a hypertrophic gene marker (BNP) and suppressing an anti-hypertrophic gene (α-MHC) in a concentration-dependent manner. Furthermore, gefitinib has been reported to induce apoptosis in cardiomyocytes via upregulating caspase 3 and P53 [22].

Currently, studies have indicated that liraglutide, a receptor agonist of glucagon-like peptide-1, possesses the ability to mitigate gefitinib-induced cardiac injury by upregulating the ERK/AKT pathway while downregulating the JNK/P38 pathway [23]. The expression of angiotensin II (AngII) and its receptors was upregulated in myocardial tissue induced by gefitinib. Valsartan inhibits the AngII type 1 receptor (AT1R), which leads to the downregulation of the JNK/P38 MAPK pathway and ultimately alleviates gefitinib-induced myocardial hypertrophy [24]. Focusing on the development of drugs that down-regulate the JNK/P38 pathway may be a promising strategy for reducing the cardiotoxicity induced by gefitinib.

##### Erlotinib

Additionally, myocardial ischemia (3.1%) and venous thromboembolism (3.9%) have also been reported among cancer patients receiving erlotinib [25, 26].

#### The second-generation EGFR-TKIs

##### Afatinib

Afatinib is a second-generation irreversible EGFR-TKI primarily targeting EGFR and human epidermal growth factor receptor 2 (HER2), which may contribute to its mechanism for inducing cardiotoxicity in NSCLC patients [27, 28]. 

There is a case report indicating that symptoms of heart failure (HF) induced by afatinib returned to normal following replacement therapy with gefitinib [29]. However, this finding contradicts previous observations regarding the induced cardiotoxicity of gefitinib, and therefore further investigation into the underlying causes is warranted.

#### The third-generation EGFR-TKIs

##### Osimertinib

Osimertinib was approved for the treatment of patients with EGFR T790M-positive NSCLC. Compared to the first- and second-generation EGFR-TKIs, osimertinib is associated with a significantly higher incidence of HF, AF, and QT interval prolongation following treatment [30, 31].

Previous studies have indicated that up to 4.9% of patients receiving osimertinib experienced grade 3 or higher cardiotoxicity, such as MI, HF, and severe mitral or tricuspid valve regurgitation [32]. Although QT interval prolongation and ventricular arrhythmias induced by osimertinib are uncommon, they can easily develop into severe arrhythmias and can be life-threatening once they occur. Currently, temporary pacemaker implantation and excessive pacing have been proposed as potential treatments for torsade de pointes induced by osimertinib [33]. In addition, AF and pericardial effusion may occur in 4% and 8.2% of patients after osimertinib treatment, and can also lead to QT interval prolongation and cardiomyopathy [34].

Clinicians are therefore advised to be able to identify tip-torsional ventricular tachycardia, to routinely monitor electrocardiograms and echocardiograms, and to avoid the use of drugs that prolong the QT interval when used in combination with other medications when treating patients with osimertinib. To more accurately identify osimertinib-induced cardiotoxicity, Li et al. employed whole-cell patch clamp technology to illustrate that osimertinib concurrently inhibited hERG (human ether-a-go-go-related gene) potassium channels, Nav1.5 sodium channels, and L-type Ca^2^⁺ channels involved in cardiomyocyte conduction, resulting in an elongation of the PR interval and QT interval [35]. This discovery establishes a foundation for future research.

##### Aumonertinib and furmonertinib

Aumonertinib (almonertinib), a novel and irreversible third-generation EGFR-TKI, was employed as a second-line treatment of NSCLC patients with the T790M mutation following the progression to EGFR-sensitive mutation therapy [36, 37]. Clinical statistics indicated that 6.1% of NSCLC patients experience QT interval prolongation after receiving aumonertinib treatment [37]. It is noteworthy that an elderly patient experienced severe HF after receiving treatment with osimertinib. However, after switching to aumonertinib, the patient's HF symptoms gradually recovered [38].

Furmonertinib is an irreversible, third-generation EGFR-TKI for the treatment of advanced or metastatic NSCLC patients with EGFR T790M mutations [39]. Treatment with furmonertinib has been associated with prolonged QT interval (9%) and AF (1%) [40].

#### Other EGFR-TKIs

##### Lapatinib

Lapatinib is a dual TKI that exerts its anti-cancer activity primarily through the inhibition of both the EGFR and the HER2 [41]. It has been estimated that lapatinib may cause a reversible reduction of LVEF in approximately 2.7% of cases [42].

Currently, anthracyclines are known to induce cardiotoxicity, and Hsu et al. have proposed that lapatinib could reverse this phenomenon by inhibiting inducible nitric oxide synthase (iNOS) [43]. However, the combination of lapatinib and doxorubicin may result in more pronounced cardiotoxicity, primarily due to lapatinib’s capacity to induce oxidative stress in cardiomyocytes through the inhibition of the PI3K/AKT signaling pathway, thereby exacerbating the cardiotoxicity induced by doxorubicin [44].

##### Neratinib

Neratinib is another HER2 inhibitor that has been indicated for the treatment of advanced HER2-positive breast cancer (BC) [45]. According to the result of the phase III clinical trial, the incidence of arrhythmia, ischemic heart disease, QT interval prolongation, and LVEF reduction in patients treated with neratinib was 3.3%, 0.7%, 2.3%, and 4.3%, respectively [46].

In conclusion, lapatinib has a relatively weak cardiotoxicity profile in comparison to other EGFR TKIs. Concerning the reduction in LVEF associated with EGFR-TKIs, clinical guidelines recommend that in cases where LVEF is greater than 50%, clinical physicians should monitor patients' echocardiograms or follow-up with them during treatment. In the event of an asymptomatic decline in LVEF during the follow-up period, the echocardiogram evaluation should be conducted within one month. If the LVEF is less than 40%, the clinician is recommended to suspend the medication and seek counseling or referral to a cardiologist for further treatment. Furthermore, the guidelines advise the monitoring of biomarkers such as CK-MB, TnT, and BNP throughout treatment, as these are more indicative of the likelihood of cardiotoxicity [47, 48].

### Anaplastic lymphoma kinase (ALK) inhibitors

Anaplastic lymphoma kinase (ALK) is a receptor tyrosine kinase that belongs to the insulin receptor superfamily. Following the binding of a ligand to its extracellular domain, ALK undergoes dimerization and subsequent autophosphorylation of its intracellular kinase domain, thereby activating the downstream oncogenic pathways, including the mitogen-activated protein kinase (MAPK) signaling cascades, which promote cancer growth and transformation [49]. Mutations in the ALK gene have been identified in a range of tumors, including NSCLC, anaplastic large cell lymphoma (ALCL), and diffuse large cell lymphoma [50–52]. The most commonly reported adverse cardiac events associated with ALK inhibitors are prolonged QT interval and bradycardia (Table 2).

**Table 2:** The cardiotoxicity of ALK/ROS1/c-Met/RET/FLT3 inhibitors

Generation	Name	Chemical structure	Targets	Approved for indications on	Cardiotoxicity	Corporation
ALK						
First-	Crizotinib (Xalkori)		ALK/ROS1/c-Met	NSCLC 08/2011	Bradycardia, pericarditis	Pfizer
Second-	Ceritinib (Zykadia)		ALK/ROS1	NSCLC 04/2014	QT interval prolongation	Novartis
	Alectinib (Alecensa)		ALK	NSCLC 12/2015	QT interval prolongation, bradycardia, pleural and pericardial effusions	Roche/Chugai
	Brigatinib (Alunbrig)		ALK/ROS1/ IGF-1R/FLT-3/EGFR	NSCLC 04/2017	Bradycardia and hypertension	Ariad
	Ensartinib		ALK	NSCLC 11/2020	Arrhythmia	BettaPharma
Third-	Lorlatinib (Lorbrena)		ALK	NSCLC 11/2018	MI	Pfizer
ROS1						
	Entrectinib (Rozlytrek)		ALK/ROS1/TRKA/B/C	Solid tumors with NTRK Fusion 08/2019	CHF, QT interval prolongation	Roche
	Larotrectinib (Vitrakvi)		TRKA/B/C	Solid tumors with NTRK Fusion 11/2018	ND	Bayer
c-MET						
	Capmatinib (Tabrecta)		c-Met	NSCLC 05/2020	ND	Novartis
	Tepotinib (Tepmetko)		c-Met	NSCLC 02/2021	ND	Novartis
	Savolitinib (Volitinib)		c-Met	NSCLC 06/2021	ND	Hutch-medicine
RET						
	Selpercatinib (Retevmo)		RET	NSCLC, MTC, Thyroid cancer 05/08/2020	Hypertension, QT interval prolongation	Loxo
	Pralsetinib (Gavreto)		RET	NSCLC, MTC, Thyroid cancer 09/04/2020	Hypertension, QT interval prolongation	Blueprint Medicines
FLT3						
First-	Midostaurin (Rydapt)		FLT3	AML 04/28/2017	Hypertension	Novartis
Second-	Gilteritinib (Xospata)		FLT3	AML 11/28/2018	QT interval prolongation	Kotobuki/Astellas
	Quizartinib (Vanflyta)		FLT3	AML 7/20/2023	QT interval prolongation	Daiichi Sankyo
Other	Pexidartinib (Turalio)		CSF1R/Kit/FLT3	Tenosynovial giant cell tumor 08/02/2019	ND	Daiichi Sankyo Inc

All data statistics from the FDA (https://www.accessdata.fda.gov)

ALK: anaplastic lymphoma kinase; IGF-1R: insulin-like growth factor-1 receptor; FLT-3: FMS-like tyrosine-3; NSCLC: non-small cell lung cancer; MI: myocardial infarction; CHF: congestive heart failure; CRC: colorectal cancer; MTC: medullary thyroid carcinoma; AML: acute myeloid leukemia; ND: No data

#### The first-generation ALK inhibitors

##### Crizotinib

Several ALK-TKIs have been granted clinical approval for the treatment of NSCLC in patients with ALK rearrangement. Crizotinib, a first-generation ALK inhibitor, is indicated for treating patients with ALK/ROS1-positive NSCLC as well as ALK-positive ALCL [53, 54].

During the treatment with crizotinib, a decrease in heart rate (below 45 bpm) may occur in most patients, leading to bradycardia [55, 56]. Conversely, a minor subset of patients experienced myocardial infarction, elevated creatinine levels, and potential sepsis [57]. The decreased heart rate may be attributed to the hyperpolarization of cardiomyocytes resulting from the inhibition of hyperpolarization-activated cyclic nucleotide-gated channel 4 by crizotinib [58]. Doherty and colleagues have reported that crizotinib suppressed hERG channels and caused cardiotoxicity by increasing ROS production, cholesterol accumulation, and activating the apoptotic cascade in cardiomyocytes in vitro [59]. Furthermore, Xu et al. have confirmed that the primary mechanism underlying the cardiotoxicity induced by crizotinib is the reduction of autophagy activity within myocardial cells. The administration of metformin has been shown to facilitate the restoration of autophagy in myocardial cells by reactivating the phosphorylation of PRKAA/AMPK (protein kinase, AMP-activated, α catalytic subunit), thereby representing a potential therapeutic strategy for alleviating crizotinib-induced cardiotoxicity. This study corroborates the assertion that metformin exerts a further novel effect [60].

Pericarditis is a frequently observed primary side effect in cancer patients undergoing treated with ALK inhibitors. The precise mechanism underlying the development of pericarditis remains unclear. The drug may exert an unidentified effect on the target, which may contribute to the onset of this adverse event [61].

#### The second-generation ALK inhibitors

Second-generation ALK inhibitors, including ceritinib, alectinib, and brigatinib, have been developed to treat cancer patients who have experienced crizotinib resistance [62–64].

##### Ceritinib and ensartinib

The prolongation of the QT interval has been reported in cancer patients treated with ceritinib [65]. In a case report, the patient received pacemaker treatment due to severe bradycardia induced by alectinib [66]. Furthermore, alectinib-induced pleural and pericardial effusions have been documented in an individual case of NSCLC patient, with resolution of effusion upon switching from alectinib to brigatinib [67]. In the ALTA trial, NSCLC patients experienced bradycardia and hypertension following brigatinib treatment [68].

Ensartinib is also a second-generation ALK inhibitor [69]. Arrhythmia has been observed in 5.9% of patients treated with ensartinib, with spontaneous resolution upon discontinuation of the medication [70].

#### The third-generation ALK inhibitors

##### Lorlatinib

Lorlatinib is a third-generation ALK inhibitor that has been demonstrated to be effective against all ALK mutations (excluding L1198F). It was approved as a first-line treatment option for advanced ALK-positive NSCLC [71, 72]. The occurrence of MI in patients with ALK-positive NSCLC who received lorlatinib was reported to be 0.7%, resulting in permanent discontinuation of the drug [73].

In conclusion, the most frequently observed adverse cardiac effects associated with ALK inhibitors are arrhythmia and pericarditis. Electrocardiograms and echocardiograms are recommended for monitoring in patients undergoing clinical application of ALK inhibitors.

### VEGFR/FGFR/PDGFR inhibitor

Tumor angiogenesis plays a pivotal role in supporting tumor cell proliferation, invasion, and metastasis [74]. Currently, the prevailing strategy for anti-angiogenic treatment is to inhibit the function of their receptors, including vascular endothelial growth factor receptor (VEGFR), platelet-derived growth factor receptor (PDGFR), and fibroblast growth factor receptor (FGFR). The most commonly used drugs in this class, such as sunitinib and sorafenib are both multi-kinase inhibitors [75]. The primary cardiotoxicity associated with this agent is hypertension, with other cardiotoxicity including decreased LVEF, congestive heart failure (CHF), and MI (Table 3).

**Table 3:** The cardiotoxicity of VEGFR/FGFR/PDGFR inhibitors

Generation	Name	Chemical structure	Targets	Approved for indications on	Cardiotoxicity	Corporation
VEGFR						
First-	Sunitinib (Sutent)		VEGFRs/ PDGFRα/β/ c-Kit/CSF1R/RET/FLT3	RCC, imatinib-resistant GIST 01/2006	hypertension, CHF, MI, decreased LVEF, QT interval prolongation, ventricular arrhythmias, transient ischemic attack	Pfizer
	Sorafenib (Nexavar)		VEGFRs/c-Kit/FLT3/RET/PDGFRβ/RAF	RCC (2005) HCC (2007) DTC (2013) Thyroid cancer (2014)	Acute coronary syndrome, hypertension, cardiac ischemia, MI, thromboembolism	Bayer
Second-	Axitinib (Inlyta)		VEGFRs	RCC after failure of sunitinib therapy 01/2012	Hypertension, HF	Pfizer
	Regorafenib (Stivarga)		VEGFRs/TIE2/FGFR1/PDGFRβ/c-KIT/RET	CRC (2012) GIST (2013) HCC (2017)	Hypertension, AF, MI, cardiac arrest	Bayer
	Tivozanib (Fotivda)		VEGFRs	RCC 02/2021	Hypertension, HF, cardiac ischemia, arterial/venous thromboembolism, bleeding	Aveo Pharms
Third-	Vandetanib (Caprelsa)		EGFR/VEGFR/RET	MTC 04/2011	QT interval prolongation, hypertension	Genzyme
FGFR						
	Erdafitinib (Balversa)		FGFRs	Urothelial carcinoma 04/2019	ND	Janssen
	Pemigatinib (Pemazyre)		FGFRs	Cholangiocarcinoma 04/2020	ND	Incyte
	InStrratinib (Truseltiq)		FGFRs	Cholangiocarcinoma 05/2021	ND	Helsinn Hlthcare
	Futibatinib (Lytgobi)		FGFRs	Intrahepatic cholangiocarcinoma 9/2022	ND	Taiho Pharmaceutical Co., Ltd
PDGFR						
	Ripretinib (Qinlock)		PDGFRα, PDGFRα mutants/Kit	GIST 05/2020	Hypertension, LV dysfunction	Deciphera
	Lenvatinib (Lenvima)		PDGFR-α/VEGFRs/ FGFRs/c-Kit/ RET	DTC (2015) Thyroid cancer (2015) RCC (2016) HCC (2018) Endometrial carcinoma (2019)	LV dysfunction, HF, hypertension	Eisai
	Avapritinib (Ayvakit)		PDGFRα, PDGFRα mutants/Kit	GIST 01/2020	ND	Blueprint Medicines
	Cabozantinib (Cabometyx, Cometriq)		BRAF/MET/VEGFRs/AXL/RET/ROS1/c-KIT/TRK/ FLT-3	MTC (2013) RCC (2016) HCC (2019)	Hypertension, bleeding*	Exelixis
	Pazopanib (Votrient)		PDGFR-β/VEGFRs/ FGFR-1/3/c-Kit/c-GSK	RCC (2009) STS (2012)	QT interval prolongation, HF, arrhythmia, ischemia, MI, hypertension	Novartis
	Nintedanib (Ofev)		VEGFR-1/2/3/PDGFR-α/β/ MDR1/BCRP/FGFR-1/3	NSCLC 2015	Hypertension	Boehringer Ingelheim
	Apatinib (Aitan)		VEGFR-2/Src/c-Kit	GC 11/2014	Hypertension	Hengrui Medicine
	Anlotinib (Focus V)		VEGFR-2/3/PDGFR-β/FGFRs	NSCLC (2018) STS (2019) SCLC (2020)	Hypertension, sinus tachycardia, and QT interval prolongation	Chia Tai Tianqing
	Fruquintinib (Elunate)		VEGFR-1/2/3	CRC 2018	Hypertension	Chi-Med/Lilly
	Surufatinib		VEGFR1/2/3, FGFR1, and CSF-1R	Neuroendocrine tumor 12/2020	Hypertension	Hutch-medicine
	Donafenib		VEGFR and PDGFR	HCC 09/2021	Hypertension	Zelgen

All data statistics from the FDA (https://www.accessdata.fda.gov)

VEGFR: vascular endothelial growth factor receptor; PDGFR: platelet-derived growth factor receptor; GSF1R: colony stimulating factor 1 receptor; RCC: renal cell carcinoma; GIST: gastrointestinal mesenchymal tumors; FLT-3: FMS-like tyrosine-3; CHF: congestive heart failure; MI: myocardial infarction; LVEF: left ventricular ejection fraction; LV: left ventricular; AF: arial fibrillation; HCC: hepatocellular carcinoma; DTC: differentiated thyroid carcinoma; CRC: colorectal cancer; MTC: medullary thyroid carcinoma; FGFR: fibroblast growth factor receptor; STS: soft tissue sarcomas; NSCLC: non-small cell lung cancer; ND: No data

#### Sunitinib

Sunitinib is a multi-kinase inhibitor that targets VEGFR-1/2/3, PDGFRα/β, c-Kit, colony-stimulating factor 1 receptor (CSF1R), RET, and FLT3, it has been approved for treating renal cell carcinoma and imatinib-resistant gastrointestinal mesenchymal tumor (GIST) [76].

The findings from a subsequent Phase III clinical trial demonstrated that treatment with sunitinib was associated with grade 3 hypertension in 8% of patients and significant reduction in LVEF in 2% of patients. Both of these adverse cardiotoxic effects were resolved after discontinuation of sunitinib [77]. Furthermore, sunitinib has been reported to induce cardiotoxicity in patients with imatinib-resistant GIST, including hypertension (47%), CHF (8%), MI (2%), and decreased LVEF (20%) [78]. Notably, the incidence of sunitinib-induced cardiotoxic events in this study was substantially higher than in previous studies, potentially due to the inclusion of a population with a high prevalence of cardiovascular diseases or a history of cardiotoxic medications, such as imatinib. The incidence of symptomatic HF was observed in 2.7% of patients after 22 days of sunitinib therapy, this was not reversible after the drug was discontinued [79, 80]. One patient experienced a severe transient ischemic attack during treatment with sunitinib [81]. Furthermore, sunitinib is also associated with a risk of ventricular arrhythmias, and it affects the QT interval in a dose-dependent manner [82].

**Fig. 3:**
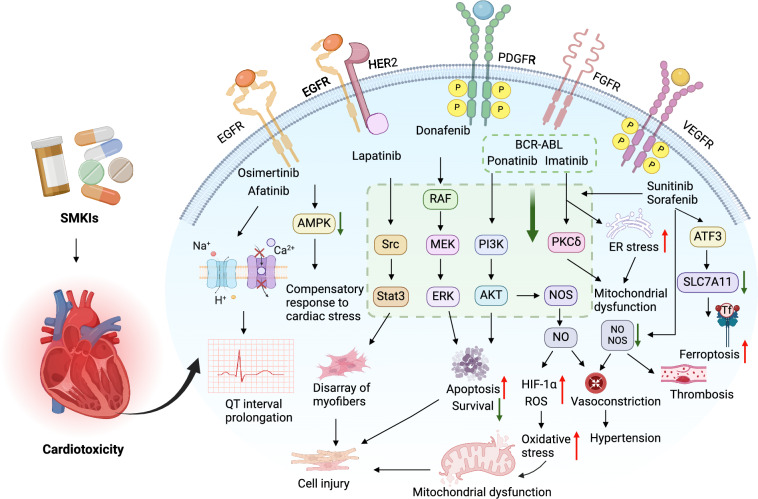
Mechanisms of SMKIs-induced cardiotoxicity. Osimertinib exhibits simultaneous inhibition of the hERG potassium channel, Nav1.5 sodium channel, and L-type Ca^2+^ channel, all of which are involved in myocardial cell conduction. This inhibition leads to the prolongation of both the PR interval and QT interval. Afatinib specifically targets the EGFR and HER2 receptors, resulting in the blockade of the PI3K/AKT pathway. This blockade subsequently leads to the accumulation of ROS and the initiation of cell apoptosis, resulting in mitochondrial damage, ultimately inducing heart failure. Moreover, lapatinib also induces oxidative stress in myocardial cells through the PI3K/AKT pathway, further contributing to cardiotoxicity. Lastly, TKIs such as sorafenib or sunitinib selectively target the VEGFR and PDGFR receptors. Hypertension is a prevalent cardiotoxicity associated with VEGFR inhibitors, primarily attributed to the inhibition of downstream signaling pathways, such as ERK/AKT, after VEGF inhibition. This inhibition results in a reduction in the production of NOS and NO by endothelial cells, leading to vasoconstriction and the eventual development of hypertension. Furthermore, elevated blood pressure imposes an increased afterload on the heart, exacerbating cardiotoxicity. Additionally, following sorafenib treatment, there is an observed elevation in ATF3 expression within myocardial cells, which inhibits the expression of SLC7A11 and promotes ferroptosis of myocardial cells, resulting in cardiotoxicity. Imatinib induces cardiotoxicity by promoting endoplasmic reticulum (ER) stress and mitochondrial dysfunction, and ponatinib induces cardiomyocyte apoptosis by inhibiting the AKT/ERK signaling pathway, leading to cardiotoxicity. EGFR: epidermal growth factor receptor; HER2: human epidermal growth factor receptor 2; ROS: reactive oxygen species; TKIs: tyrosine kinase inhibitors; PDGFR: platelet-derived growth factor receptor; NOS: nitric oxide synthase; NO: nitric oxide; ER: endoplasmic reticulum

The mechanisms contributing to sunitinib-induced cardiotoxicity include the inhibition of VEGFR, PDGFR, and AMPK expression, hypoxic stress, the activation of endothelin-1, and ferroptosis (Fig. 3). And the downregulation of VEGFR or PDGFR expressions may potentially result in a reduction in the density of coronary capillaries, thereby limiting the contraction or expansion of the left ventricle [83]. Cardiomyocytes have been demonstrated to express PDGFRs, and elevated levels of PDGF have been associated with cardiomyocyte viability. Nevertheless, the inhibition of PDGFRs has the potential to induce apoptosis and cause cardiotoxicity [84].

It has been established that the AMPK signaling pathway promotes the survival of cardiomyocytes. It can be inferred that sunitinib may exert its cardiotoxic effect by inhibiting AMPK activity. This is corroborated by the observation that transfection with an adenoviral vector expressing AMPK is capable of significantly alleviating sunitinib-induced cardiomyocyte death. Additionally, trimetazidine is an antianginal drug that has been demonstrated to mitigate cardiotoxicity by activating the AMPK/mTOR/autophagy signaling pathway [85].

Hypertension represents a significant risk factor for the development of left ventricular dysfunction and HF in patients receiving sunitinib. It has been confirmed that sunitinib-induced hypertension is primarily associated with the activation of endothelin-1 and the inhibition of the renin-angiotensin system [86, 87].

Furthermore, sunitinib activates the hypoxia-responsive gene HIF-1α, which can result in cardiomyocyte damage and a range of cardiomyopathies such as CHF [88]. The results of various animal studies indicate that thalidomide, FGF2 mRNA, endothelin receptor antagonist macitentan, sacubitril/valsartan, and AMPK activator may be employed as therapeutic methods for heart protection [83, 89–91].

Moreover, sunitinib induces cardiomyocyte apoptosis and cardiotoxicity through autophagic degradation of CCN2 (cellular communication network factor 2), and HMGB1 (high mobility group box 1) serves as an important regulatory factor for cardiotoxicity. Consequently, the inhibition of HMGB1 can alleviate the cardiotoxicity of sunitinib [92].

Recent studies have shown that sunitinib induces cardiotoxicity by promoting oxidative stress and Nrf2-dependent ferroptosis [93].

Therefore, it is imperative that clinicians meticulously monitor the patients’ blood pressure and LVEF throughout treatment, particularly in individuals with a history of hypertension or coronary artery disease.

#### Sorafenib

Sorafenib is the first clinically approved multikinase inhibitor for anti-angiogenesis therapy, exhibiting potent inhibitory effects on VEGFR-1/2/3, c-Kit, FLT3, RET, PDGFRβ, and RAF [94]. It has been approved for treating unresectable hepatocellular carcinoma, advanced renal cell carcinoma, and differentiated thyroid carcinoma [95].

However, it is important to note that VEGFR inhibitors have the potential to induce hypertension and thromboembolic events. It has been reported that sorafenib causes acute coronary syndrome (including MI) in patients [96]. Furthermore, in a phase III clinical trial involving renal cancer patients, sorafenib was shown to induce a significantly higher incidence of grade 3 or 4 hypertension in 16.8% of patients and cardiac ischemia or MI in 2.6% of patients [97, 98]. Overall, the incidence of sorafenib-induced hypertension during sorafenib treatment did not exceed 20% of patients. Moreover, a meta-analysis of clinical trials revealed that 1.7% and 1.3% of patients experienced thromboembolic events following treatment with sorafenib and sunitinib, respectively [99].

The potential cardiotoxic mechanism of sorafenib has been the subject of investigation. The cardiotoxicity induced by sorafenib mainly involves the loss of rapidly accelerated fibrosarcoma 1 (RAF1), promotion of myocardial cell ferroptosis, and inhibition of endothelial nitric oxide synthase (NOS) and nitric oxide (NO) in endothelial cells.

It’s established that sorafenib exerts a potent inhibitory effect on the activity of the RAF kinase family, which is a member of the mitogen-activated protein kinase (MAPK) kinases [100]. The key member of this family, RAF1, is a proto-oncogene product and a Ser/Thr kinase that may play an important role in normal cell growth and oncogenic transformation. In the context of oxidative stress-induced injury, RAF1 has been observed to inhibit the pro-apoptotic kinases ASK1 (apoptosis signal-regulating kinase 1) and MST2 (mammalian Ste20-like kinase 2), thereby preventing excessive tissue damage. The knockdown of the RAF1 gene has been demonstrated to result in an increase in cardiomyocyte apoptosis, a decline in myocardial contractility, and ultimately cardiac dilation [101, 102].

A series of recent studies have demonstrated that sorafenib additionally induces cardiotoxicity by triggering ferroptosis in myocardial cells. The expression of ATF3 in myocardial cells in patients treated with sorafenib was found to be significantly up-regulated. The results of both in vivo and in vitro experiments corroborate the hypothesis that ATF3 inhibits the expression of SLC7A11 and promotes ferroptosis in myocardial cells, ultimately inducing cardiotoxicity [103]. Jiang et al. have confirmed that ATF4 can reverse sorafenib-induced cardiotoxicity by upregulating SLC7A11 expression and inhibiting ferroptosis [104]. Moreover, ferrostatin-1, a ferroptosis inhibitor, attenuated sorafenib-induced cardiac fibrosis by reducing KLF11-mediated ferroptosis [105].

In conclusion, targeted ferroptosis may be a promising treatment option for preventing sorafenib-induced cardiotoxicity.

The induction of hypertension by sunitinib and sorafenib may be attributed to their inhibitory effect on NOS (nitric oxide synthase) and NO production by endothelial cells, leading to vasoconstriction and ultimately elevated blood pressure [106]. Additionally, the reduction of NO contributes to thrombosis. Similarly, sunitinib and sorafenib may lead to thrombosis by disrupting endothelial cell integrity and exposing prethrombotic phospholipids [107]. VEGFR inhibitors promote platelet aggregation leading to thrombosis by activating the platelet FcδRIIa immunoglobulin G receptors through binding to VEGF [108].

#### Axitinib

Axitinib, a second-generation VEGFR inhibitor with higher selectivity than sorafenib, was approved for treating advanced renal cell carcinoma following the failure of sunitinib therapy [109]. In a phase III trial of axitinib in 359 patients with advanced renal cell carcinoma, 42% of patients experienced all-grade hypertension, with 17% of these patients presenting with grade 3 or above hypertension [110]. When hypertension is poorly controlled, it can lead to serious cardiovascular events. It is therefore incumbent upon clinicians to monitor the blood pressure of patients on axitinib closely and adjust the drug dose accordingly.

#### Regorafenib

Regorafenib, a multi-targeted kinase inhibitor, is indicated for patients treated for CRC, GIST, and hepatocellular carcinoma (HCC) [111, 112]. As is the case with other multikinase inhibitors, regorafenib may cause cardiac adverse events. In a phase 3 trial of GIST patients treated with regorafenib, the incidence of all grade, grade 3, and grade 4 hypertension was 48.5%, 22.7%, and 0.8%, respectively, with one patient experiencing cardiac arrest [113]. In another CONCUR trial, AF and MI were reported in two CRC patients treated with regorafenib [114].

#### Vandetanib

Vandetanib is an EGFR, VEGFR, and RET inhibitor, which has been approved for treating medullary thyroid cancer [115]. Among the most frequent adverse events associated with vandetanib were hypertension and extended QT interval. Zang et al. reported that 12% and 18% of patients encountered all-grade and high-grade QT interval prolongation in thyroid cancer patients [116]. In a meta-analysis of 3154 patients, it was found that 24.2% of patients experienced all-grade hypertension, and 6.4% experienced high-grade hypertension during vandetanib treatment [117].

#### Nintedanib and others

Nintedanib was initially approved for the treatment of idiopathic pulmonary fibrosis, however, recent evidence has demonstrated its efficacy in the treatment of metastatic esophageal and gastric cancer. The primary targets of nintedanib are the VEGFR-1/2/3, FGFR1/2, and PDGFR α/β. A clinical trial reported that 15% of 32 patients with metastatic esophageal gastric cancer developed grade 3 hypertension after receiving treatment with nintedanib [118]. Other multikinase inhibitors, including ripretinib, cabozantinib, and pazopanib, have also demonstrated cardiotoxicities, such as QT interval prolongation, hypertension, and MI [119–122].

Similar to other VEGFR inhibitors, apatinib has been found to induce hypertension. In a phase III clinical trial, it was observed that 35.23% of patients developed hypertension (below grade 4) after two weeks of apatinib administration [123]. Research has shown that apatinib induces hypertension by activating the RhoA/ROCK (rho-associated coiled-coil-containing kinase) pathway, which can be reversed by the ROCK inhibitor Y-27632 [124]. The principal cardiotoxicities associated with anlotinib encompass hypertension (67.35%), sinus tachycardia (35.71%), and QT interval prolongation (26.19%) [125].

Hypertension has similarly been identified as significant cardiotoxicity in multi-kinase inhibitors, including fruquintinib, surufatinib, and donafenib [126–128]. Consequently, it is advised that patients’ blood pressure be monitored regularly to prevent hypertensive crises.

It is noteworthy that the risk of cardiotoxic events triggered by multi-kinase inhibitors is typically associated with the pre-existing cardiovascular disease of the patients. It is hypothesized that TKI-induced hypertension may exacerbate cardiac stress. It is therefore important to obtain a patient’s cardiovascular history before treatment and to monitor blood pressure during treatment. It has been proposed that pharmacological intervention with appropriate antihypertensive agents, such as angiotensin-converting enzyme (ACE) inhibitors or angiotensin II receptor blockers (ARBs), may be beneficial for cancer patients experiencing hypertension [129].

## Non-receptor tyrosine kinase inhibitors

### BCR-ABL inhibitors

BCR-ABL is a fusion gene generated primarily by the Philadelphia (Ph) chromosome translocation. It encodes the oncoprotein p210 Bcr-Abl1, which has aberrant tyrosine kinase activity and drives the proliferation of leukemia cells [130]. Imatinib, dasatinib, and nilotinib are the major clinically approved BCR-ABL inhibitors for treating chronic myeloid leukemia (CML). Inhibition of the BCR-ABL tyrosine kinase can prolong patient survival but is often associated with cardiac toxic events (Table 4).

**Table 4:** The cardiotoxicity of BCR-ABL inhibitors

Generation	Name	Chemical structure	Targets	Approved for indications on	Cardiotoxicity	Corporation
First-	Imatinib (Glivec)		BCR-ABL/PDGFR/c-Kit	2001 (CML) 2003 (GIST) 2006 (ALL)	LV dysfunction, CHF	Novartis
Second-	Dasatinib (Sprycel)		BCR-ABL/PDGFR/c-Kit	Ph^+^ ALL, CML, and as a therapeutic strategy after imatinib resistance 06/2006	QT interval prolonged, CHF, LV dysfunction, hypertension	Bristol-Myers Squibb
	Nilotinib (Tasigna)		BCR-ABL/PDGFR/c-Kit/CSF-1R	Ph^+^ CML patients who are resistant to imatinib 10/2007	Atherosclerosis, AMI, atrial flutter and fibrillation	Novartis
	Bosutinib (Bosulif)		BCR-ABL/PDGFR/c-Kit/CSF-1R	Ph^+^ CML patients who are resistant to imatinib 09/2012	HF	Pfizer
	Flumatinib		BCR-ABL	CML 11/2019	ND	Hansoh Pharma
**Third-**	Ponatinib (Iclusig)		BCR-ABL/PDGFR-α/VEGFR-2/ FGFR-1/Src/FLT3/c-Kit	imatinib-resistant patients 12/2012	Arrhythmia, hypertension, angina, MI, atherosclerosis, atrial fibrillation and atrial flutter, venous/arterial thrombosis*, CHF*	Incyte/Takeda
	Asciminib (Scemblix)		BCR-ABL	Ph^+^ CML patients with concomitant T315I mutation 10/2021	Induced weaker cardiotoxicity	Novartis
	Olverembatinib		BCR-ABL	Ph^+^ CML, ALL 11/2021	Hypertension, pericardial effusion, outdoor systolic phase, AF, and supraventricular extraventricular contractions	Ascentage Pharma

All data statistics from the FDA (https://www.accessdata.fda.gov)

PDGFR: platelet-derived growth factors receptor; CML: chronic myeloid leukemia; GIST: gastrointestinal stromal tumor; ALL: acute lymphoblastic leukemia; LV: left ventricular; CHF: congestive heart failure; CSF-1R: colony-stimulating factor 1 receptor; AMI: acute myocardial infarction; VEGFR-2: vascular endothelial growth factor receptor 2; FGFR-1: fibroblast growth factor receptor-1; FLT3: fms-like tyrosine kinase 3

#### Imatinib

Imatinib is the first-generation BCR-ABL inhibitor and also the first small-molecule TKI developed for targeted cancer therapy [131]. It was approved for treating CML in 2001. It has been reported that imatinib therapy is inevitably associated with cardiac adverse events [132].

The initial finding of imatinib-induced CHF was considered a rare adverse event, with an incidence of only 0.7% [133, 134]. Using human and rat cardiomyocyte models, Kerkela et al. found that imatinib activated endoplasmic reticulum stress, which triggered reduced mitochondrial function and cell death [135]. Imatinib was demonstrated to elevate protein kinase Cδ (PKCδ) expression and induce apoptosis in cardiomyocytes [136].

N-terminal pro-B-type natriuretic peptide (NT-proBNP) is a diagnostic test for contractile dysfunction in left ventricular cardiomyocytes. The plasma level of brain natriuretic peptide was found to be markedly elevated in two patients with GIST who had developed HF following treatment with imatinib. In light of these findings, Park et al. proposed the use of BNP as a diagnostic or predictive marker for imatinib-induced HF [137].

Furthermore, studies in mouse models have demonstrated that the mitochondrial damage and myocardial cell death induced by imatinib are age-dependent [138]. Consequently, it’s recommended that the heart function of elderly patients should be monitored more closely during imatinib treatment.

A variety of research strategies have been developed to reduce the cardiotoxicity associated with imatinib therapy. Marslin et al. developed a stable poly(lactide-co-glycolide) nanoparticle formulation of imatinib to reduce its cardiotoxicity through the sustained release of the drug [139]. As the cardiotoxic effect of imatinib was derived from the inhibition of the c-Abl kinase, imatinib has been structurally modified to reduce its Bcr-Abl inhibition while increasing the inhibitory effect on c-Kit and an additional target JNK [140]. The modified compounds were found to maintain c-Kit inhibition and exhibit a potent anticancer effect in a mouse GIST model with a marked reduction in cardiotoxicity [140]. It may therefore be postulated that JNK activation by oxidative stress is related to imatinib-induced cardiotoxicity, and that inhibition of the JNK signaling pathway can reduce mitochondrial dysfunction and cell apoptosis [141].

In conclusion, the clinical applications of imatinib in cancer treatment should be approached with caution. The current guideline recommends that patients with risk factors for coronary artery disease or HF should have their left ventricular function assessed before initiation of imatinib therapy [142].

#### Dasatinib

Second-generation BCR-ABL inhibitors, such as dasatinib, nilotinib, and bosutinib, have been developed due to the inevitable emergence of drug resistance [143].

Dasatinib is a multikinase inhibitor that targets c-KIT, PDGFR-α/β, and BCR-ABL. It is indicated for treating adult and pediatric Ph^+^ ALL (acute lymphoblastic leukemia) and CML and it is also commonly considered a therapeutic strategy following imatinib resistance [144].

However, dasatinib is known to induce more severe cardiotoxicity than imatinib. CHF with prolonged QT interval and pulmonary hypertension was reported in 2.2% of patients after dasatinib treatment [145]. As dasatinib-induced pulmonary hypertension may be due to hypoxia-induced attenuation of the vasoconstrictor response, it could be resolved after dasatinib discontinuation [146].

#### Ponatinib

Ponatinib, the third-generation BCR-ABL inhibitor, is particularly effective against the BCR-ABL T315I mutation, which confers resistance to imatinib [147].

However, ponatinib induces the most severe cardiotoxicity of all BCR-ABL inhibitors. According to a study evaluating CML patients diagnosed from 2002 to 2017, cardiotoxic events occurred in approximately 1 in 5 patients, including angina in 15.6% of patients, atherosclerosis in 13.7% of patients, and AF or atrial flutter in 8.7% of patients, which are potentially life-threatening adverse effects [145]. In addition, ponatinib-induced cardiotoxicity events also include hypertension (13.3%), pericardial effusion (8.5%), outer systolic phase (4.2%), and supraventricular extraventricular contractions (3%) [148].

Recently, ponatinib was shown to induce excessive inflammatory responses in cardiomyocytes by activating the S100A8/A9-NLRP3-IL-1β signaling pathway, which could be counteracted by dexamethasone [149]. Singh et al. proposed that ponatinib induced apoptosis in cardiomyocytes by inhibiting the AKT/ERK signaling pathway [150]. Hnatiuk et al. developed novel ponatinib analogs that retain antitumor activity against CML but with significantly reduced cardiotoxicity [151, 152]. Research suggests that aspirin may be effective in the reduction of adverse events such as ponatinib-induced thrombosis [153].

ROCK inhibition has been shown to reverse endothelial dysfunction induced by dasatinib and ponatinib, thereby alleviating cardiotoxicity. Thus, ROCK has been suggested to have great potential as a molecular target to address BCR-ABL inhibitor-associated cardiotoxicity [154].

Based on the above research findings, the risk–benefit ratio of ponatinib should be carefully assessed before selecting it for CML patients. Once ponatinib is initiated, it is important to closely monitor the patient’s cardiopulmonary function.

### Bruton’s tyrosine kinase (BTK) inhibitors

Bruton’s tyrosine kinase (BTK), a member of the TEC family of non-receptor protein tyrosine kinases, plays a critical role in driving oncogenic signaling to support leukemic cell proliferation and survival in many B-cell malignancies [155]. In recent years, the FDA has approved several BTK inhibitors, including ibrutinib, acalabrutinib, and zanubrutinib, for treating patients with CLL (chronic lymphocytic leukemia) and lymphoma [156, 157]. Although BTK inhibitors have revolutionized the treatment strategy for B-cell malignancies, induction of AF, hypertension, and ventricular arrhythmias has been demonstrated by BTK inhibitors in clinical use [158, 159] (Table 5).

**Table 5:** The cardiotoxicity of BTK inhibitors

Generation	Name	Chemical structure	Targets	Approved for indications on	Cardiotoxicity	Corporation
First-	Ibrutinib (Imbruvica)		BTK	MCL (2013) CLL (2014) WM (2015) SLL (2016) MZL (2017)	Ventricular arrhythmias, hypertension	AbbVie/Johnson & Johnson
Second-	Acalabrutinib (Calquence)		BTK	MCL 10/2017	AF, hypertension	AstraZeneca
	Zanubrutinib (Brukinsa)		BTK	MCL 11/2019	AF, hypertension	BeiGene
	Pirtobrutinib (Jaypirca)		BTK	MCL 01/2023	AF	Eli Lilly
	Orelabrutinib		BTK	MCL, CLL, SLL 12/2020	Hypertension, QT interval prolongation	Innocarepharma

All data statistics from the FDA (https://www.accessdata.fda.gov)

BTK: Bruton’s tyrosine kinase; MCL: mantle cell lymphoma; CLL: chronic lymphocytic leukemia; WM: Waldenstrom’s macroglobulinemia; SLL: small lymphocytic lymphoma; MZL: marginal zone lymphoma; AF: atrial fibrillation; HF: heart failure; ND: No data

#### Ibrutinib

Ibrutinib, the first irreversible BTK inhibitor, binds to the Cys-481 residue in the ATP binding site of BTK and prevents autophosphorylation. Ibrutinib has received FDA approval for the treatment of newly diagnosed CLL, although the manufacturer has voluntarily withdrawn its indication for use in adult patients with mantle cell lymphoma [156, 160].

A comprehensive analysis of the current data suggests that the prevalence of ibrutinib-induced AF is in the range of 5–20%, and is predominantly observed in patients aged 65 years or older and in those with an underlying cardiovascular disease [161]. In addition, ibrutinib-induced AF occurred over approximately 3–5 months, but the symptoms of AF were resolved in most patients within approximately one week of discontinuing the drug [162].

Ibrutinib-induced AF was mediated by an off-target effect of the drug on the C-terminal SRC kinase (CSK) [163]. Administration of ibrutinib in a BTK kinase-deficient mouse model was found to induce AF, left atrial enlargement, and myocardial fibrosis, which surprisingly was not found in the treatment with the acalabrutinib mouse model. Furthermore, the knockdown of CSK also induced the development of AF, implying that inhibition of CSK expression may contribute to the induction of AF by ibrutinib [163].

On the other hand, ibrutinib not only upregulates the expression of calmodulin kinase 2 (CaMKII) but also increases the phosphorylation of ryanodine receptor 2 (RyR2) within the endoplasmic reticulum of cardiomyocytes, which ultimately leads to ectopic electrical activity of intracellular calcium ions [164]. BTK has also been reported to activate its downstream PI3K, which is a key molecule associated with cardiac rhythm. And after PI3K is inhibited, AF is more likely to occur [165, 166].

A series of studies have documented that patients undergoing treatment with ibrutinib have experienced ventricular arrhythmias (VA) and sudden cardiac death, despite the majority of these individuals lacking a prior history of cardiovascular conditions [167–169]. Although VA is relatively rare in the context of ibrutinib-induced cardiotoxicity, it warrants significant attention from clinicians due to its status as one of the most severe arrhythmias associated with ibrutinib use [170, 171]. The mechanism of VA induced by ibrutinib mainly involves calcium imbalance, electrophysiological instability, and inhibition of the activity of AMPK and PI3K/AKT signaling pathways.

Ibrutinib has been reported to induce VA through dysregulation of calcium release and repolarization in cardiomyocytes [172]. Research has shown that ibrutinib inhibited the activation of the AMPK pathway, which further suppressed sarcoplasmic reticulum Ca^2+^-ATPase 2a (SERCA2a) levels, leading to calcium handling abnormalities and ultimately to uneven repolarization of cardiomyocytes, resulting in VA. The AMPK agonist 5-aminoimidazole-4-carboxamide-1-β-ribofuranoside prophylaxis could potentially attenuate ibrutinib-induced VA [173, 174]. Recent studies have demonstrated that metformin mitigates ibrutinib-induced VA, primarily by enhancing the activity of the AMPK and PI3K/AKT signaling pathways [175]. And, there was a significant increase in the risk of VA during follow-up in patients treated with ibrutinib. Consequently, it is imperative to closely monitor patient changes, and discontinue the drug if necessary [167]. In addition, Du et al. reported that ibrutinib induced ventricular fibrillation by reducing the amplitude of calcium peaks in an elderly rat model of spontaneous hypertension, but this phenomenon was not observed in a young rat model [176].

It is common for patients treated with ibrutinib to discontinue treatment because the drug has been significantly associated with an increased incidence of AF, thromboembolism, or stroke, as well as an increased risk of bleeding due to administration of anticoagulants for AF management. Therefore, acalabrutinib, a newer generation BTK inhibitor has been developed as a replacement for ibrutinib [177, 178].

#### Acalabrutinib

Acalabrutinib, the second-generation BTK inhibitor with greater selectivity, has been approved by the FDA for use in previously treated mantle cell lymphoma and newly diagnosed CLL [179, 180]. Similar to the earlier generation BTK inhibitors, acalabrutinib has also been reported to cause AF, hypertension, and VA.

A recent follow-up study of patients with B-cell malignancies (n = 1063) receiving acalabrutinib showed that 8 of these patients experienced VA. This was the first report of a VA event associated with the clinical use of acalabrutinib [181]. The most common cardiotoxic event induced by acalabrutinib was AF, with an incidence of 3–4% [182–184]. In the ELEVATE-RR trial, AF occurred in 9% and 16% of the 533 CLL patients who received acalabrutinib and ibrutinib, respectively. Moreover, ibrutinib-induced AF led to treatment discontinuation in approximately 3.4% of patients, whereas no patients in the acalabrutinib group discontinued treatment due to AF [185, 186]. These results suggested that acalabrutinib had a lower propensity to induce cardiotoxicity than ibrutinib. Therefore, acalabrutinib could be considered to treat CLL patients who have discontinued ibrutinib due to its toxicity [187].

The mechanism underlying the induction of AF by acalabrutinib is not clear. Unlike ibrutinib, ascalabrutinib did not inhibit CSK in mouse models and the pathophysiology leading to ascalabrutinib-induced AF remains to be elucidated [163].

#### Zanubrutinib

Zanubrutinib, another novel irreversible BTK inhibitor, is also approved for treating mantle cell lymphoma and demonstrates more potent anticancer activity compared to ibrutinib [188]. AF was observed in 2.5% of CLL patients receiving zanubrutinib in the phase III ALPINE trial [189].

Hypertension is a recognized adverse effect associated with both ibrutinib and zanubrutinib, suggesting that hypertension may be a class effect of BTK inhibitors. Specifically, hypertension is a documented side effect of ibrutinib treatment [190, 191]. Long-term analyses indicate that 28% of patients treated with ibrutinib developed hypertension of grade 3 or higher [192]. Furthermore, data suggest that 78.3% of patients experienced new or worsening hypertension during ibrutinib treatment, a rate significantly higher than that predicted by the Framingham Heart Study [193]. These findings collectively imply that ibrutinib may contribute to the increase in blood pressure. Subsequent studies revealed that 48.9% of patients developed hypertension following treatment with acalabrutinib [194]. Results from a phase III clinical trial demonstrated that the incidence of hypertension induced by zanbrutinib was lower than that induced by ibrutinib, with 5% of patients experiencing ≥ grade 3 hypertension [195, 196].

The precise mechanism by which BTK inhibitors induce hypertension remains unclear. Should patients develop hypertension during treatment with BTK inhibitors, clinicians are advised to intervene promptly in accordance with established guidelines for antihypertensive medications, such as ACEIs and ARBs. It is uncommon for patients to discontinue treatment due to hypertension-related side effects, except in instances of grade 3 or higher hypertension. However, the dosage of BTK inhibitors may be adjusted once blood pressure is stabilized [194].

BTK inhibitors remain a mainstay of treatment for B-cell lymphomas. There is an unmet medical need to develop novel BTK inhibitors with minimal off-target effects. When choosing a drug selection, clinicians must reduce the potential risk of cardiotoxicity and closely monitor the cardiac adverse events to ensure patients receive the greatest therapeutic benefit [197].

## Animal model of SMKIs-induced cardiotoxicity

Currently, several animal models are used to observe or study the mechanism of SMKI-induced cardiotoxicity. We have summarized some common in vivo animal models in Table 6.

**Table 6:** The available models to investigate drug cardiotoxicity from SMKIs in vivo

Target	Drug	Dose	Time	Animals	indications	Mechanism	Treatment	Refs.
EGFR	Gefitinib	30 mg/kg/day	2W	Adult male Sprague–Dawley rats	Cardiac hypertrophy	Increasing PTEN and FoxO3a gene expression	CYP1A1 inhibitor (α-naphthoflavone)	[21]
	Gefitinib	30 mg/kg/day	3W	Wistar albino rats	Cardiac hypertrophy	Upregulating caspase 3 and P53	ND	[22]
	Gefitinib	30 mg/kg/day, oral gavage	3W	Male Wistar albino rats	Cardiac hypertrophy	Downregulating ERK/AKT, upregulating JNK/P38	Liraglutide	[23]
	Gefitinib	30 mg/kg/day	4W	Male Wistar albino rats	Cardiac hypertrophy	Increasing the level of AngII and its receptors	AT1R blocking valsartan	[24]
	Lapatinib	10 mg/kg/day, *i.p.*		Male, C57BL/6 mice, − 8w		Increasing iNOS expression and pronouncing production of NO	iNOS inhibition	[43]
				Human pluripotent stem cell-derived cardiomyocytes (HpsC-CMs)				
ALK	Crizotinib	100 mg/kg/day, intragastric administration	6W	C57BL/6J mice 7–9w	Left ventricular dysfunction, myocardial injury cardiomyocyte apoptosis and mitochondrial injury	Inhibition of PRKAA/AMPK phosphorylation	Metformin	[60]
VEGFR	Sunitinib	26.7 mg/kg	8 days	Male Wistar-Kyoto rats	Blood pressure (hypertension)	Activation of the endothelin-1 system, suppression of the renin-angiotensin system	Sunitinib withdrawal	[86]
	Sunitinib	50 mg/kg/day, intragastric administration	6W	C57BL/6 mice, 6W	Hypertension, increase in ejection fraction and cardiac fibrosis	Increasing in cardiac uptake of [18F] fluorodeoxyglucose	Co-administration of macitentan	[89]
	Sunitinib	40 mg/kg/day, intragastric administration	32 days	C57BL/6 mice or athymic nude mice BALB/c-nude, 5–6W	Cardiomyocyte death and cardiotoxicity	The autophagic degradation of CCN2	HMGB1-specific inhibitor glycyrrhizic acid	[92]
	Sunitinib	10 mg/kg/day, intragastric administration	2W	C57BL/6J male mice, 8–10W	Tachycardia	Reduced the expression of Nrf2, HO-1, NQO1, GPX4 and FTH1, enhanced the TfR expression (Nrf2-dependent ferroptosis)	ND	[93]
	Sunitinib	20 mg/kg/day, intragastric administration	2W	C57BL/6J male mice, 8-10W	Bradycardia			
	Sunitinib	25 mg/kg, three times a week, intragastric administration	4W	Male Wistar albino rats, 8W	Cardiac fibrosis	Upregulation of NF-ΚB/Wnt/β-catenin/SOX9 pathway	Sacubitril/valsartan	[90]
	Sunitinib	40 mg/kg/day, intragastric administration	4W	Male 129S1/SvImJ mice	Hypertension, left ventricular dysfunction	inhibition of AMPK/mTOR/autophagy pathway	Trimetazidine	[85]
	Sunitinib	50 mg/kg/day, intragastric administration	5 days	Female C57BL/6, 15 weeks	Myocardial fibrosis	Upregulates glycolysis and downregulates oxidative metabolism in cardiac mitochondria	Macitentan	[91]
	Sorafenib	30 mg/kg/day, *i.p.*	2W	Male C57BL/6 mice, 6W	loss in cardiac contractile function	Upregulated ferroptosis-related protein expression: PTGS2, Slc7a11 and GPX4 were decreased	Ferrostatin-1	[103]
	Sorafenib	50 mg/kg twice daily, intragastric administration	3W	8-week-old male C57BL/6 mice	cardiomyocytes hypertrophy	SOR evoked ferroptosis of cardiomyocytes, and the cytosolic lipid ROS accumulation in ferroptosis cardiomyocytes triggered ER stress and UPR	ATF4 overexpression	[104]
	Sorafenib	30 mg/kg/day, *i.p.*	2W	C57BL/6 mice	Heart injury	KLF11 promoted ferroptosis by suppressing transcription of FSP1	Targeting ferroptosis	[105]
	Apatinib	50 mg/kg/day, intragastric administration	4W	Female BALB/C nude mice (4–6 weeks old, 20 ± 5 g)	Hypertension	Mediating the significant upregulation of RhoA, ROCK1 and ROCK2 expression	ROCK1 inhibitor Y27632	[124]
BCR-ABL	Ponatinib	APOE−/− TACE, 15 mg/kg/day, intragastric administration	6W	8-week-old male and female C57Bl/6J mice	Cardiac inflammation	Activating the S100A8/A9-TLR4-NLRP3-IL-1β signaling pathway	glucocorticoid dexamethasone or NLRP3 inhibitor (CY-09) or S100A9 inhibitor (paquinimod)	[149]
	Ponatinib	10 μM		Zebrafish	Cardiomyocytes death	Inhibiting cardiac prosurvival signaling pathways AKT and ERK, induces cardiomyocyte apoptosis	Neuregulin-1β	[150]
	Ponatinib	10 μM		Tg (cmlc2: GFP) transgenic zebrafish	Cardiac edema, abnormal heart structure, low heart rate, cardiac cell death, and thrombosis	AKT, MAPK, MAP3K and TEK were downregulated, COX-1 was up-regulated	COX-1 inhibitor aspirin	[153]
	Ibrutinib	25 mg/kg/day, *i.p.*	4W	C57BL/6J mice, 3- to 4-month-old	Atrial fibrillation	Inhibition of C-Terminal Src Kinas	ND	[163]
	Ibrutinib	25 mg/kg/day, orally	4W	C57BL/6J mice	Atrial fibrillation	Inducing structural remodeling and calcium dysregulation in the atrium	ND	[164]
	Ibrutinib	10 mg/kg/day, orally	4W	Male Sprague–Dawley rats, age range: 10–14 months	Ventricular arrhythmias	Impairment of myocardial AMPK activity	AMPK activator 5‐aminoimidazole‐4‐carboxamide‐1‐β‐d‐ribofuranoside	[174]
	Ibrutinib	30 mg/kg/day, orally	4W	Male C57BL/6J mice aged 3 months	Ventricular arrhythmias	Decreasing AMPK and PI3K-AKT pathway activity	Metformin	[175]

*I.p.*: intraperitoneal injection; AT1R: AngII type 1 receptor; InoS: Inducible nitric oxide synthase; CCN2: Cellular communication network factor 2; HMGB1: High mobility group box 1; FSP1: Ferroptosis suppressor protein 1; TLR4: Toll-like receptor 4; NLRP3: NLR family pyrin domain-containing 3; COX-1: Cyclooxygenase-1

## Current therapeutic strategies

Treatment strategies for SMKIs-induced cardiotoxicity currently emphasize dose reduction or discontinuation of the offending agent, switching to alternative medications, and symptomatic management.

Patients diagnosed with cancer should be comprehensively assessed before treatment with SMKIs, with particular attention to cardiovascular risk factors such as hyperlipidemia and previous history of cardiovascular disease. In high-risk patients, pre-treatment ECG, echocardiography, and blood pressure monitoring are essential to effectively manage potential adverse events. Consultation with cardiac oncologists may also be warranted to develop safer and more detailed treatment strategies. Continuous monitoring during treatment is essential for the early detection of cardiotoxicity, and targeted interventions, such as electrocardiography, echocardiography, and markers of myocardial injury can serve as effective monitoring. Cardiac oncology guidelines provide tailored monitoring recommendations based on individual patient risk factors [198].

Preventing the occurrence of cardiotoxicity is a primary goal, as it can significantly reduce the risk of adverse effects in patients. The American Heart Association and the American Association of Cardiovascular and Pulmonary Rehabilitation have identified exercise, dietary modification, weight management, and psychological counseling, among other interventions, as fundamental components of a cardiac rehabilitation program [199–201]. Additionally, pharmacological preventive strategies may include the administration of ACE inhibitors (ACEIs), angiotensin II receptor blockers, β-blockers, or statins in high-risk hypertensive patients before starting treatment [202]. β-blockers such as nebivolol are considered the preferred agents for the treatment of left ventricular dysfunction associated with SMKIs. Furthermore, metoprolol or diltiazem may be employed to reduce QT interval prolongation associated with pazopanib therapy. Follow-up during the later stages of treatment is also critical to understanding delayed cardiotoxicity and providing effective solutions for patients.

In specific cases, it’s important to analyze each situation individually. For example, in patients with multiple cardiovascular risk factors, clinicians should choose drugs with less cardiotoxicity. In patients with T315I mutations, ponatinib should be discontinued in favor of ascimitinib to reduce unnecessary toxic side effects. Additionally, BTK inhibitors are not recommended in individuals with ventricular arrhythmia, uncontrolled hypertension, or crisis. The individual circumstances of each patient must be assessed along with their clinical status to formulate an appropriate treatment plan through multidisciplinary consultation [203].

In conclusion, clinicians should proactively address SMKI-induced cardiotoxicity by implementing strategies such as preventive measures, pre-treatment assessments, monitoring throughout therapy, and timely interventions aimed at minimizing the risk of cardiotoxicity. Multidisciplinary consultation can further enhance the development of optimized therapeutic strategies, thus individualized approaches are critical in reducing patients’ exposure to cardiotoxicity. This strategy can effectively minimize the risk without compromising overall therapeutic outcomes and ultimately benefit the patient’s prognosis.

## Conclusion

Although small molecule kinase inhibitors (SMKIs) are central to cancer therapy, the incidence of cardiotoxicity events cannot be overlooked. These include QT interval prolongation, hypertension, arrhythmias, HF, myocardial ischemia, or infarction.

The mechanism of SMKIs-induced cardiotoxicity involves several key aspects, such as signaling pathway inhibition, cardiac ion channel disturbances, reactive oxygen species (ROS) accumulation, and vascular endothelial dysfunction. SMKIs targeting kinases (e.g., EGFR, HER2, c-Kit, and PDGFR), such as gefitinib, ibrutinib, and sorafenib, may interfere with the signaling pathways required for cardiomyocyte survival (e.g., PI3K/Akt, ERK), leading to apoptosis or necrosis of cardiomyocytes. Another example is imatinib, which affects myocardial repair mechanisms by inhibiting c-Kit and PDGFR. Furthermore, cardiac ion channel disturbances, including hERG potassium channel blockade and calcium homeostasis imbalance, have been identified as mechanisms by which SMKIs induce cardiotoxicity. For instance, the inhibition of hERG channels caused by osimertinib and sunitinib has been observed to prolong myocardial repolarization, resulting in QT interval prolongation and the subsequent development of ventricular tachycardia. Moreover, ibrutinib has been shown to interfere with sarcoplasmic reticulum calcium-regulated proteins (e.g., SERCA2a), leading to calcium overloading or release abnormalities, which, in turn, can induce arrhythmias or contractile dysfunction. SMKIs-induced cardiotoxicity is also associated with reactive oxygen species (ROS) accumulation. For instance, crizotinib induced the production of ROS, which have been demonstrated to inflict damage to myocardial cell membranes, proteins, and DNA, thereby triggering oxidative damage. Inhibition of fatty acid/glucose metabolism in cardiomyocytes can also induce cardiotoxicity, and drugs targeting the AMPK pathway, such as crizotinib, sunitinib, and ibrutinib, may interfere with myocardial energy substrate utilization, leading to metabolic remodeling. Finally, vascular endothelial dysfunction can lead to vascular and microcirculatory disorders, such as sunitinib inhibition of the VEGF pathway leading to apoptosis of endothelial cells, reducing NO production, and triggering myocardial ischemia or hypertension.

Several cardiotoxicity events may be life-threatening to the patient, clinicians are therefore challenged with the dual task of minimizing cardiotoxicity without compromising cancer treatment. A comprehensive understanding of SMKIs-induced cardiotoxicity is essential for clinicians to improve patient outcomes and quality of life. Clinicians can intervene to prevent life-threatening cardiotoxicity from SMKIs. Interventions consist primarily of early monitoring, targeted interventions, and individualized medications. For example, certain SMKIs have a high susceptibility to cardiotoxicity, necessitating a comprehensive cardiac assessment before administration. In susceptible populations, such as patients with heart failure, clinicians regularly monitor cardiac-related tests, including electrocardiogram (QT interval), echocardiogram (LVEF), and biomarkers (troponin, BNP). The vast majority of patients have been shown to recover from SMKI-induced cardiotoxicity after discontinuation of the drug or treatment. In addition to the aforementioned measures, the use of targeted drugs such as dexrazoxane, which acts as a ROS scavenger, and β-blockers, which act as antiarrhythmics, has been shown to mitigate drug-induced cardiotoxicity. Finally, the use of individualized medications by clinicians can help avoid cardiotoxic events that may be caused by drug combinations.

This review summarizes only the cardiotoxicity of SMKIs that has been reported to date. As more SMKIs are developed for the treatment of tumors, more cardiotoxic adverse effects will be reported. For the SMKIs newly approved in recent years, more extensive clinical trials recruiting larger cohorts of subjects will be needed to reveal the extent of their cardiotoxicity. Although we have summarized the mechanistic work investigating the cardiotoxicity induced by some SMKIs, the detailed mechanisms leading to some cardiotoxic adverse events have not been fully elucidated and remain to be explored.

## Data Availability

No datasets were generated or analysed during the current study.

## Abbreviations

ACE: Angiotensin-converting enzyme
AF: Atrial fibrillation
ALCL: Anaplastic large cell lymphoma
ALK: Anaplastic lymphoma kinase
ALL: Acute lymphocytic lymphoma
AML: Acute myeloid leukemia
ARB: Angiotensin II receptor blocker
BC: Breast cancer
BNP: Brain natriuretic peptide
BTK: Bruton's tyrosine kinase
CaMKII: Calmodulin kinase 2
CHF: Congestive heart failure
CLL: Chronic lymphocytic leukemia
c-Met: Cellular-mesenchymal-epithelial transition factor
CML: Chronic myeloid leukemia
CNS: Central nervous system
CRC: Colorectal cancer
CSF1R: Colony-stimulating factor 1 receptor
CSK: C-terminal SRC kinase
ECG: Electrocardiogram
EF: Ejection fraction
EGFR: Epidermal growth factor receptor
ER: Estrogen receptor
FDA: Food and Drug Administration
FGF: Fibroblast growth factor
FLT3: FMS-like tyrosine kinase 3
GIST: Gastrointestinal mesenchymal tumors
HCC: Hepatocellular carcinoma
HER2: Human epidermal growth factor receptor 2
HF: Heart failure
HGF: Hepatocyte growth factor
iNOS: Inducible nitric oxide synthase
LVEF: Left ventricular ejection fraction
MI: Myocardial infarction
NO: Nitric oxide
NOS: Nitric oxide synthase
NSCLC: Non-small cell lung cancer
NT-proBNP: N-terminal pro-B-type natriuretic peptide
PDGFR: Platelet-derived growth factor receptor
PKCδ: Protein kinase Cδ
PTC: Papillary thyroid carcinoma
SERCA2a: Sarcoplasmic reticulum Ca^2+^-ATPase 2a
SLL: Small lymphocytic lymphoma
SMKIs: Small-molecule kinase inhibitors
TKI: Tyrosine kinase inhibitor
TME: Tumor microenvironment
VEGFR: Vascular endothelial growth factor receptor

## References

[1] Xia C, Dong X, Li H, Cao M, Sun D, He S, et al. Cancer statistics in china and United States, 2022: profiles, trends, and determinants. Chin Med J. 2022;135:584–90. 35143424 10.1097/CM9.0000000000002108 PMC8920425

[2] Bray F, Laversanne M, Weiderpass E, Soerjomataram I. The ever-increasing importance of cancer as a leading cause of premature death worldwide. Cancer. 2021;127:3029–30. 34086348 10.1002/cncr.33587

[3] Sung H, Ferlay J, Siegel RL, Laversanne M, Soerjomataram I, Jemal A, et al. Global cancer statistics 2020: globocan estimates of incidence and mortality worldwide for 36 cancers in 185 countries. CA Cancer J Clin. 2021;71:209–49. 33538338 10.3322/caac.21660

[4] Bedard PL, Hyman DM, Davids MS, Siu LL. Small molecules, big impact: 20 years of targeted therapy in oncology. Lancet. 2020;395:1078–88. 32222192 10.1016/S0140-6736(20)30164-1

[5] Savage DG, Antman KH. Imatinib mesylate–a new oral targeted therapy. N Engl J Med. 2002;346:683–93. 11870247 10.1056/NEJMra013339

[6] Desai A, Reddy NK, Subbiah V. Top advances of the year: precision oncology. Cancer. 2023;129:1634–42. 36946766 10.1002/cncr.34743

[7] Zhong L, Li Y, Xiong L, Wang W, Wu M, Yuan T, et al. Small molecules in targeted cancer therapy: advances, challenges, and future perspectives. Signal Transduct Target Ther. 2021;6:201. 34054126 10.1038/s41392-021-00572-w PMC8165101

[8] Smith CEP, Prasad V. Targeted cancer therapies. Am Fam Physician. 2021;103:155–63. 33507053

[9] Ayala-Aguilera CC, Valero T, Lorente-Macías Á, Baillache DJ, Croke S, Unciti-Broceta A. Small molecule kinase inhibitor drugs (1995–2021): medical indication, pharmacology, and synthesis. J Med Chem. 2022;65:1047–131. 34624192 10.1021/acs.jmedchem.1c00963

[10] Roskoski R Jr. Properties of fda-approved small molecule protein kinase inhibitors: a 2024 update. Pharmacol Res. 2024;200: 107059. 38216005 10.1016/j.phrs.2024.107059

[11] Jain D, Russell RR, Schwartz RG, Panjrath GS, Aronow W. Cardiac complications of cancer therapy: pathophysiology, identification, prevention, treatment, and future directions. Curr Cardiol Rep. 2017;19:36. 28374177 10.1007/s11886-017-0846-x

[12] Suh E, Stratton KL, Leisenring WM, Nathan PC, Ford JS, Freyer DR, et al. Late mortality and chronic health conditions in long-term survivors of early-adolescent and young adult cancers: a retrospective cohort analysis from the Childhood Cancer Survivor Study. Lancet Oncol. 2020;21:421–35. 32066543 10.1016/S1470-2045(19)30800-9 PMC7392388

[13] Schwartz RG, Jain D, Storozynsky E. Traditional and novel methods to assess and prevent chemotherapy-related cardiac dysfunction noninvasively. J Nucl Cardiol. 2013;20:443–64. 23572315 10.1007/s12350-013-9707-1

[14] Guardiola S, Varese M, Sánchez-Navarro M, Giralt E. A third shot at EGFR: new opportunities in cancer therapy. Trends Pharmacol Sci. 2019;40:941–55. 31706618 10.1016/j.tips.2019.10.004

[15] Li X, Lin Y, Lin S, Huang J, Ruan Z. Advancements in understanding cardiotoxicity of EGFR- TKIs in non-small cell lung cancer treatment and beyond. Front Pharmacol. 2024;15:1404692. 39211774 10.3389/fphar.2024.1404692 PMC11357958

[16] Huang J, Meng L, Yang B, Sun S, Luo Z, Chen H. Safety profile of epidermal growth factor receptor tyrosine kinase inhibitors: a disproportionality analysis of FDA adverse event reporting system. Sci Rep. 2020;10:4803. 32179761 10.1038/s41598-020-61571-5 PMC7075865

[17] Yamaguchi K, Kanazawa S, Kinoshita Y, Muramatsu M, Nomura S. Acute myocardial infarction with lung cancer during treatment with gefitinib: the possibility of gefitinib-induced thrombosis. Pathophysiol Haemost Thromb. 2005;34:48–50. 16293986 10.1159/000088548

[18] Lynch DR Jr, Kickler TS, Rade JJ. Recurrent myocardial infarction associated with gefitinib therapy. J Thromb Thrombol. 2011;32:120–4. 10.1007/s11239-010-0539-4 21184253

[19] Omori S, Oyakawa T, Naito T, Takahashi T. Gefitinib-induced cardiomyopathy in epidermal growth receptor-mutated NSCLC. J Thorac Oncol. 2018;13:e207–8. 30244856 10.1016/j.jtho.2018.05.020

[20] Truell JS, Fishbein MC, Figlin R. Myocarditis temporally related to the use of gefitinib (Iressa). Arch Pathol Lab Med. 2005;129:1044–6. 16048398 10.5858/2005-129-1044-MTRTTU

[21] Alhoshani A, Alanazi FE, Alotaibi MR, Attwa MW, Kadi AA, Aldhfyan A, et al. EGFR inhibitor gefitinib induces cardiotoxicity through the modulation of cardiac PTEN/Akt/Foxo3a pathway and reactive metabolites formation: in vivo and in vitro rat studies. Chem Res Toxicol. 2020;33:1719–28. 32370496 10.1021/acs.chemrestox.0c00005

[22] Korashy HM, Attafi IM, Ansari MA, Assiri MA, Belali OM, Ahmad SF, et al. Molecular mechanisms of cardiotoxicity of gefitinib in vivo and in vitro rat cardiomyocyte: role of apoptosis and oxidative stress. Toxicol Lett. 2016;252:50–61. 27084042 10.1016/j.toxlet.2016.04.011

[23] AlAsmari AF, Ali N, AlAsmari F, AlAnazi WA, AlShammari MA, Al-Harbi NO, et al. Liraglutide attenuates gefitinib-induced cardiotoxicity and promotes cardioprotection through the regulation of MAPK/NF-κB signaling pathways. Saudi Pharm J. 2020;28:509–18. 32273812 10.1016/j.jsps.2020.03.002 PMC7132601

[24] Alanazi WA, Alhamami HN, Alharbi M, Alhazzani K, Alanazi AS, Alsanea S, et al. Angiotensin II type 1 receptor blockade attenuates gefitinib-induced cardiac hypertrophy via adjusting angiotensin II-mediated oxidative stress and JNK/P38 MAPK pathway in a rat model. Saudi Pharm J. 2022;30:1159–69. 36164571 10.1016/j.jsps.2022.06.020 PMC9508643

[25] Moore MJ, Goldstein D, Hamm J, Figer A, Hecht JR, Gallinger S, et al. Erlotinib plus gemcitabine compared with gemcitabine alone in patients with advanced pancreatic cancer: a phase III trial of the national cancer Institute of Canada Clinical Trials Group. J Clin Oncol. 2007;25:1960–6. 17452677 10.1200/JCO.2006.07.9525

[26] Zaborowska-Szmit M, Krzakowski M, Kowalski DM, Szmit S. Cardiovascular complications of systemic therapy in non-small-cell lung cancer. J Clin Med. 2020;9:1268. 32349387 10.3390/jcm9051268 PMC7287714

[27] Maione P, Sacco PC, Sgambato A, Casaluce F, Rossi A, Gridelli C. Overcoming resistance to targeted therapies in NSCLC: current approaches and clinical application. Ther Adv Med Oncol. 2015;7:263–73. 26327924 10.1177/1758834015595048 PMC4543855

[28] Kühn B. ERBB2 inhibition and heart failure. N Engl J Med. 2013;368:875–6. 23445110 10.1056/NEJMc1215887

[29] Nuvola G, Dall’Olio FG, Melotti B, Sperandi F, Ardizzoni A. Cardiac toxicity from afatinib in EGFR-mutated NSCLC: a rare but possible side effect. J Thorac Oncol. 2019;14:e145–6. 31235038 10.1016/j.jtho.2019.02.027

[30] Anand K, Ensor J, Trachtenberg B, Bernicker EH. Osimertinib-induced cardiotoxicity: a retrospective review of the FDA adverse events reporting system (FAERS). JACC CardioOncol. 2019;1:172–8. 34396179 10.1016/j.jaccao.2019.10.006 PMC8352117

[31] Waliany S, Zhu H, Wakelee H, Padda SK, Das M, Ramchandran K, et al. Pharmacovigilance analysis of cardiac toxicities associated with targeted therapies for metastatic NSCLC. J Thorac Oncol. 2021;16:2029–39. 34418561 10.1016/j.jtho.2021.07.030

[32] Kaira K, Ogiwara Y, Naruse I. Occurrence of ventricular fibrillation in a patient with lung cancer receiving osimertinib. J Thorac Oncol. 2020;15:e54–5. 32216947 10.1016/j.jtho.2019.11.029

[33] Zhang Y, Wang X, Pan Y, Du B, Nanthakumar K, Yang P. Overdrive pacing in the acute management of osimertinib-induced ventricular arrhythmias: a case report and literature review. Front Cardiovasc Med. 2022;9: 934214. 36247453 10.3389/fcvm.2022.934214 PMC9557095

[34] Mok TS, Wu YL, Ahn MJ, Garassino MC, Kim HR, Ramalingam SS, et al. Osimertinib or platinum-pemetrexed in EGFR T790M-positive lung cancer. N Engl J Med. 2017;376:629–40. 27959700 10.1056/NEJMoa1612674 PMC6762027

[35] Li P, Tian X, Wang G, Jiang E, Li Y, Hao G. Acute osimertinib exposure induces electrocardiac changes by synchronously inhibiting the currents of cardiac ion channels. Front Pharmacol. 2023;14:1177003. 37324483 10.3389/fphar.2023.1177003 PMC10267729

[36] Nagasaka M, Zhu VW, Lim SM, Greco M, Wu F, Ou SI. Beyond osimertinib: the development of third-generation EGFR tyrosine kinase inhibitors for advanced EGFR+ NSCLC. J Thorac Oncol. 2021;16:740–63. 33338652 10.1016/j.jtho.2020.11.028

[37] Lu S, Wang Q, Zhang G, Dong X, Yang CT, Song Y, et al. Efficacy of aumolertinib (HS-10296) in patients with advanced EGFR T790M+ NSCLC: updated post-national medical products administration approval results from the apollo registrational trial. J Thorac Oncol. 2022;17:411–22. 34801749 10.1016/j.jtho.2021.10.024

[38] Zhang Q, Liu H, Yang J. Aumolertinib effectively reduces clinical symptoms of an EGFR l858r-mutant non-small cell lung cancer case coupled with osimertinib-induced cardiotoxicity: case report and review. Front Endocrinol. 2022;13: 833929. 10.3389/fendo.2022.833929 PMC9170288 35677717

[39] Deeks ED. Furmonertinib: first approval. Drugs. 2021;81:1775–80. 34528187 10.1007/s40265-021-01588-w

[40] Shi Y, Chen G, Wang X, Liu Y, Wu L, Hao Y, et al. Furmonertinib (AST2818) versus gefitinib as first-line therapy for Chinese patients with locally advanced or metastatic EGFR mutation-positive non-small-cell lung cancer (FURLONG): a multicentre, double-blind, randomised phase 3 study. Lancet Respir Med. 2022;10:1019–28. 35662408 10.1016/S2213-2600(22)00168-0

[41] Voigtlaender M, Schneider-Merck T, Trepel M. Lapatinib. Recent Results Cancer Res. 2018;211:19–44. 30069757 10.1007/978-3-319-91442-8_2

[42] Choi HD, Chang MJ. Cardiac toxicities of lapatinib in patients with breast cancer and other HER2-positive cancers: a meta-analysis. Breast Cancer Res Treat. 2017;166:927–36. 28825152 10.1007/s10549-017-4460-9

[43] Hsu WT, Huang CY, Yen CYT, Cheng AL, Hsieh PCH. The HER2 inhibitor lapatinib potentiates doxorubicin-induced cardiotoxicity through iNOS signaling. Theranostics. 2018;8:3176–88. 29930721 10.7150/thno.23207 PMC6010982

[44] Sun L, Wang H, Xu D, Yu S, Zhang L, Li X. Lapatinib induces mitochondrial dysfunction to enhance oxidative stress and ferroptosis in doxorubicin-induced cardiomyocytes via inhibition of PI3K/AKT signaling pathway. Bioengineered. 2022;13:48–60. 34898356 10.1080/21655979.2021.2004980 PMC8805895

[45] Burstein HJ, Sun Y, Dirix LY, Jiang Z, Paridaens R, Tan AR, et al. Neratinib, an irreversible ErbB receptor tyrosine kinase inhibitor, in patients with advanced ErbB2-positive breast cancer. J Clin Oncol. 2010;28:1301–7. 20142587 10.1200/JCO.2009.25.8707

[46] Saura C, Oliveira M, Feng YH, Dai MS, Chen SW, Hurvitz SA, et al. Neratinib plus capecitabine versus lapatinib plus capecitabine in HER2-positive metastatic breast cancer previously treated with ≥ 2 HER2-directed regimens: phase III NALA trial. J Clin Oncol. 2020;38:3138–49. 32678716 10.1200/JCO.20.00147 PMC7499616

[47] Chatsiproios D. Safety profile and clinical recommendations for the use of lapatinib. Breast Care. 2010;5:16–21. 20847928 10.1159/000285776 PMC2931096

[48] Dent SF, Kikuchi R, Kondapalli L, Ismail-Khan R, Brezden-Masley C, Barac A, et al. Optimizing cardiovascular health in patients with cancer: a practical review of risk assessment, monitoring, and prevention of cancer treatment-related cardiovascular toxicity. Am Soc Clin Oncol Educ Book. 2020;40:1–15. 32213102 10.1200/EDBK_286019

[49] Hallberg B, Palmer RH. The role of the alk receptor in cancer biology. Ann Oncol. 2016;27(Suppl 3):iii4–15. 27573755 10.1093/annonc/mdw301

[50] Ferreri AJ, Govi S, Pileri SA, Savage KJ. Anaplastic large cell lymphoma, alk-positive. Crit Rev Oncol Hematol. 2012;83:293–302. 22440390 10.1016/j.critrevonc.2012.02.005

[51] Beltran B, Castillo J, Salas R, Quiñones P, Morales D, Hurtado F, et al. ALK-positive diffuse large B-cell lymphoma: Report of four cases and review of the literature. J Hematol Oncol. 2009;2:11. 19250532 10.1186/1756-8722-2-11 PMC2651189

[52] Remon J, Pignataro D, Novello S, Passiglia F. Current treatment and future challenges in ROS1- and ALK-rearranged advanced non-small cell lung cancer. Cancer Treat Rev. 2021;95: 102178. 33743408 10.1016/j.ctrv.2021.102178

[53] Shaw AT, Yeap BY, Solomon BJ, Riely GJ, Gainor J, Engelman JA, et al. Effect of crizotinib on overall survival in patients with advanced non-small-cell lung cancer harbouring ALK gene rearrangement: a retrospective analysis. Lancet Oncol. 2011;12:1004–12. 21933749 10.1016/S1470-2045(11)70232-7 PMC3328296

[54] Mossé YP, Voss SD, Lim MS, Rolland D, Minard CG, Fox E, et al. Targeting ALK with crizotinib in pediatric anaplastic large cell lymphoma and inflammatory myofibroblastic tumor: a Children’s Oncology Group Study. J Clin Oncol. 2017;35:3215–21. 28787259 10.1200/JCO.2017.73.4830 PMC5617123

[55] Ou SH, Tong WP, Azada M, Siwak-Tapp C, Dy J, Stiber JA. Heart rate decrease during crizotinib treatment and potential correlation to clinical response. Cancer. 2013;119:1969–75. 23505007 10.1002/cncr.28040

[56] Ou SH, Azada M, Dy J, Stiber JA. Asymptomatic profound sinus bradycardia (heart rate ≤45) in non-small cell lung cancer patients treated with crizotinib. J Thorac Oncol. 2011;6:2135–7. 22088989 10.1097/JTO.0b013e3182307e06

[57] Schöffski P, Sufliarsky J, Gelderblom H, Blay JY, Strauss SJ, Stacchiotti S, et al. Crizotinib in patients with advanced, inoperable inflammatory myofibroblastic tumours with and without anaplastic lymphoma kinase gene alterations (European Organisation for Research and Treatment of Cancer 90101 CREATE): a multicentre, single-drug, prospective, non-randomised phase 2 trial. Lancet Respir Med. 2018;6:431–41. 29669701 10.1016/S2213-2600(18)30116-4

[58] Zhang Z, Huang TQ, Nepliouev I, Zhang H, Barnett AS, Rosenberg PB, et al. Crizotinib inhibits hyperpolarization-activated cyclic nucleotide-gated channel 4 activity. Cardiooncology. 2017;3:1–8. 28217366 10.1186/s40959-017-0020-z PMC5310672

[59] Doherty KR, Wappel RL, Talbert DR, Trusk PB, Moran DM, Kramer JW, et al. Multi-parameter in vitro toxicity testing of crizotinib, sunitinib, erlotinib, and nilotinib in human cardiomyocytes. Toxicol Appl Pharmacol. 2013;272:245–55. 23707608 10.1016/j.taap.2013.04.027

[60] Xu Z, Pan Z, Jin Y, Gao Z, Jiang F, Fu H, et al. Inhibition of PRKAA/AMPK (Ser485/491) phosphorylation by crizotinib induces cardiotoxicity via perturbing autophagosome-lysosome fusion. Autophagy. 2023. 10.1080/15548627.2023.2259216. 37733896 10.1080/15548627.2023.2259216 PMC10813574

[61] Niimura T, Miyata K, Hamano H, Nounin Y, Unten H, Yoshino M, et al. Cardiovascular toxicities associated with anaplastic lymphoma kinase inhibitors: a disproportionality analysis of the WHO Pharmacovigilance Database (VigiBase). Drug Saf. 2023;46:545–52. 37106270 10.1007/s40264-023-01300-9

[62] Shaw AT, Kim DW, Mehra R, Tan DS, Felip E, Chow LQ, et al. Ceritinib in ALK-rearranged non-small-cell lung cancer. N Engl J Med. 2014;370:1189–97. 24670165 10.1056/NEJMoa1311107 PMC4079055

[63] Shaw AT, Gandhi L, Gadgeel S, Riely GJ, Cetnar J, West H, et al. Alectinib in ALK-positive, crizotinib-resistant, non-small-cell lung cancer: a single-group, multicentre, phase 2 trial. Lancet Oncol. 2016;17:234–42. 26708155 10.1016/S1470-2045(15)00488-X PMC4752892

[64] Kim DW, Tiseo M, Ahn MJ, Reckamp KL, Hansen KH, Kim SW, et al. Brigatinib in patients with crizotinib-refractory anaplastic lymphoma kinase-positive non-small-cell lung cancer: a randomized, multicenter phase II trial. J Clin Oncol. 2017;35:2490–8. 28475456 10.1200/JCO.2016.71.5904

[65] Shah RR, Morganroth J. Update on cardiovascular safety of tyrosine kinase inhibitors: with a special focus on QT interval, left ventricular dysfunction and overall risk/benefit. Drug Saf. 2015;38:693–710. 26008987 10.1007/s40264-015-0300-1

[66] Pruis MA, Veerman GDM, Hassing HC, Lanser DAC, Paats MS, van Schaik RHN, et al. Cardiac toxicity of alectinib in patients with ALK+ lung cancer: outcomes of cardio-oncology follow-up. JACC CardioOncol. 2023;5:102–13. 36875894 10.1016/j.jaccao.2022.09.006 PMC9982223

[67] Ulhoi MP, Sorensen BS, Meldgaard P. Alectinib-induced pleural and pericardial effusions in ALK-positive NSCLC. Case Rep Oncol. 2021;14:1323–7. 34720935 10.1159/000518081 PMC8525301

[68] Brigatinib MA. First global approval. Drugs. 2017;77:1131–5. 28597393 10.1007/s40265-017-0776-3

[69] Zhu VW, Ou SI. Ensartinib (x-396), an approved alk inhibitor, falls out as a clinically relevant ROS1 inhibitor. J Thorac Oncol. 2021;16:1778–81. 34715999 10.1016/j.jtho.2021.07.005

[70] Horn L, Wang Z, Wu G, Poddubskaya E, Mok T, Reck M, et al. Ensartinib vs crizotinib for patients with anaplastic lymphoma kinase-positive non-small cell lung cancer: a randomized clinical trial. JAMA Oncol. 2021;7:1617–25. 34473194 10.1001/jamaoncol.2021.3523 PMC8414368

[71] Solomon BJ, Bauer TM, Ignatius Ou SH, Liu G, Hayashi H, Bearz A, et al. Post hoc analysis of lorlatinib intracranial efficacy and safety in patients with ALK-positive advanced non-small-cell lung cancer from the phase III CROWN study. J Clin Oncol. 2022;40:3593–602. 35605188 10.1200/JCO.21.02278 PMC9622589

[72] Solomon BJ, Besse B, Bauer TM, Felip E, Soo RA, Camidge DR, et al. Lorlatinib in patients with ALK-positive non-small-cell lung cancer: results from a global phase 2 study. Lancet Oncol. 2018;19:1654–67. 30413378 10.1016/S1470-2045(18)30649-1

[73] U.S. Food and Drug Administration. Lorbrena (lorlatinib) tablets. 2018. https://www.accessdata.fda.gov/drugsatfda_docs/label/2018/210868s000lbl.pdf. Accessed 2 Nov 2018.

[74] Jiang X, Wang J, Deng X, Xiong F, Zhang S, Gong Z, et al. The role of microenvironment in tumor angiogenesis. J Exp Clin Cancer Res. 2020;39:204. 32993787 10.1186/s13046-020-01709-5 PMC7526376

[75] Qi S, Deng S, Lian Z, Yu K. Novel drugs with high efficacy against tumor angiogenesis. Int J Mol Sci. 2022;23:6934. 35805939 10.3390/ijms23136934 PMC9267017

[76] Goodman VL, Rock EP, Dagher R, Ramchandani RP, Abraham S, Gobburu JV, et al. Approval summary: sunitinib for the treatment of imatinib refractory or intolerant gastrointestinal stromal tumors and advanced renal cell carcinoma. Clin Cancer Res. 2007;13:1367–73. 17332278 10.1158/1078-0432.CCR-06-2328

[77] Motzer RJ, Hutson TE, Tomczak P, Michaelson MD, Bukowski RM, Rixe O, et al. Sunitinib versus interferon alfa in metastatic renal-cell carcinoma. N Engl J Med. 2007;356:115–24. 17215529 10.1056/NEJMoa065044

[78] Chu TF, Rupnick MA, Kerkela R, Dallabrida SM, Zurakowski D, Nguyen L, et al. Cardiotoxicity associated with tyrosine kinase inhibitor sunitinib. Lancet. 2007;370:2011–9. 18083403 10.1016/S0140-6736(07)61865-0 PMC2643085

[79] Khakoo AY, Kassiotis CM, Tannir N, Plana JC, Halushka M, Bickford C, et al. Heart failure associated with sunitinib malate: a multitargeted receptor tyrosine kinase inhibitor. Cancer. 2008;112:2500–8. 18386829 10.1002/cncr.23460

[80] Telli ML, Witteles RM, Fisher GA, Srinivas S. Cardiotoxicity associated with the cancer therapeutic agent sunitinib malate. Ann Oncol. 2008;19:1613–8. 18436521 10.1093/annonc/mdn168

[81] van der Veldt AA, Boven E, Helgason HH, van Wouwe M, Berkhof J, de Gast G, et al. Predictive factors for severe toxicity of sunitinib in unselected patients with advanced renal cell cancer. Br J Cancer. 2008;99:259–65. 18594533 10.1038/sj.bjc.6604456 PMC2480961

[82] Bello CL, Mulay M, Huang X, Patyna S, Dinolfo M, Levine S, et al. Electrocardiographic characterization of the QTc interval in patients with advanced solid tumors: pharmacokinetic- pharmacodynamic evaluation of sunitinib. Clin Cancer Res. 2009;15:7045–52. 19903787 10.1158/1078-0432.CCR-09-1521

[83] Yang Y, Bu P. Progress on the cardiotoxicity of sunitinib: prognostic significance, mechanism and protective therapies. Chem Biol Interact. 2016;257:125–31. 27531228 10.1016/j.cbi.2016.08.006

[84] Hsieh PC, MacGillivray C, Gannon J, Cruz FU, Lee RT. Local controlled intramyocardial delivery of platelet-derived growth factor improves postinfarction ventricular function without pulmonary toxicity. Circulation. 2006;114:637–44. 16894033 10.1161/CIRCULATIONAHA.106.639831

[85] Yang Y, Li N, Chen T, Zhang C, Liu L, Qi Y, et al. Trimetazidine ameliorates sunitinib-induced cardiotoxicity in mice via the AMPK/mTOR/autophagy pathway. Pharm Biol. 2019;57:625–31. 31545912 10.1080/13880209.2019.1657905 PMC6764339

[86] Kappers MH, van Esch JH, Sluiter W, Sleijfer S, Danser AH, van den Meiracker AH. Hypertension induced by the tyrosine kinase inhibitor sunitinib is associated with increased circulating endothelin-1 levels. Hypertension. 2010;56:675–81. 20733093 10.1161/HYPERTENSIONAHA.109.149690

[87] Kappers MH, de Beer VJ, Zhou Z, Danser AH, Sleijfer S, Duncker DJ, et al. Sunitinib-induced systemic vasoconstriction in swine is endothelin mediated and does not involve nitric oxide or oxidative stress. Hypertension. 2012;59:151–7. 22124432 10.1161/HYPERTENSIONAHA.111.182220

[88] Moslehi J, Minamishima YA, Shi J, Neuberg D, Charytan DM, Padera RF, et al. Loss of hypoxia-inducible factor prolyl hydroxylase activity in cardiomyocytes phenocopies ischemic cardiomyopathy. Circulation. 2010;122:1004–16. 20733101 10.1161/CIRCULATIONAHA.109.922427 PMC2971656

[89] Sourdon J, Facchin C, Certain A, Viel T, Robin B, Lager F, et al. Sunitinib-induced cardiac hypertrophy and the endothelin axis. Theranostics. 2021;11:3830–8. 33664864 10.7150/thno.49837 PMC7914356

[90] Mohamad HE, Askar ME, Shaheen MA, Baraka NM, Mahmoud YK. Sacubitril/valsartan alleviates sunitinib-induced cardiac fibrosis and oxidative stress via improving TXNIP/TRX system and downregulation of NF-ĸB/Wnt/β-catenin/SOX9 signaling. Int Immunopharmacol. 2024;132: 111963. 38560962 10.1016/j.intimp.2024.111963

[91] Sourdon J, Lager F, Viel T, Balvay D, Moorhouse R, Bennana E, et al. Cardiac metabolic deregulation induced by the tyrosine kinase receptor inhibitor sunitinib is rescued by endothelin receptor antagonism. Theranostics. 2017;7:2757–74. 28824714 10.7150/thno.19551 PMC5562214

[92] Xu Z, Jin Y, Gao Z, Zeng Y, Du J, Yan H, et al. Autophagic degradation of CCN2 (cellular communication network factor 2) causes cardiotoxicity of sunitinib. Autophagy. 2022;18:1152–73. 34432562 10.1080/15548627.2021.1965712 PMC9196717

[93] Li D, Song C, Song C, Tian X, Zhang H, Zhang J, et al. Sunitinib induces cardiotoxicity through modulating oxidative stress and Nrf2-dependent ferroptosis in vitro and in vivo. Chem Biol Interact. 2024;388: 110829. 38101598 10.1016/j.cbi.2023.110829

[94] Wilhelm SM, Carter C, Tang L, Wilkie D, McNabola A, Rong H, et al. BAY 43-9006 exhibits broad spectrum oral antitumor activity and targets the RAF/MEK/ERK pathway and receptor tyrosine kinases involved in tumor progression and angiogenesis. Cancer Res. 2004;64:7099–109. 15466206 10.1158/0008-5472.CAN-04-1443

[95] Llovet JM, Ricci S, Mazzaferro V, Hilgard P, Gane E, Blanc JF, et al. Sorafenib in advanced hepatocellular carcinoma. N Engl J Med. 2008;359:378–90. 18650514 10.1056/NEJMoa0708857

[96] Koehler VF, Berg E, Adam P, Weber GL, Pfestroff A, Luster M, et al. Real-world efficacy and safety of multi-tyrosine kinase inhibitors in radioiodine refractory thyroid cancer. Thyroid. 2021;31:1531–41. 34405734 10.1089/thy.2021.0091

[97] Escudier B, Eisen T, Stadler WM, Szczylik C, Oudard S, Siebels M, et al. Sorafenib in advanced clear-cell renal-cell carcinoma. N Engl J Med. 2007;356:125–34. 17215530 10.1056/NEJMoa060655

[98] Gridelli C, Maione P, Del Gaizo F, Colantuoni G, Guerriero C, Ferrara C, et al. Sorafenib and sunitinib in the treatment of advanced non-small cell lung cancer. Oncologist. 2007;12:191–200. 17296815 10.1634/theoncologist.12-2-191

[99] Choueiri TK, Schutz FA, Je Y, Rosenberg JE, Bellmunt J. Risk of arterial thromboembolic events with sunitinib and sorafenib: a systematic review and meta-analysis of clinical trials. J Clin Oncol. 2010;28:2280–5. 20351323 10.1200/JCO.2009.27.2757

[100] Kyriakis JM, Force TL, Rapp UR, Bonventre JV, Avruch J. Mitogen regulation of c-Raf-1 protein kinase activity toward mitogen-activated protein kinase-kinase. J Biol Chem. 1993;268:16009–19. 8340422

[101] Muslin AJ. Role of raf proteins in cardiac hypertrophy and cardiomyocyte survival. Trends Cardiovasc Med. 2005;15:225–9. 16182133 10.1016/j.tcm.2005.06.008

[102] Yamaguchi O, Watanabe T, Nishida K, Kashiwase K, Higuchi Y, Takeda T, et al. Cardiac-specific disruption of the c-raf-1 gene induces cardiac dysfunction and apoptosis. J Clin Invest. 2004;114:937–43. 15467832 10.1172/JCI20317 PMC518660

[103] Li Y, Yan J, Zhao Q, Zhang Y, Zhang Y. Atf3 promotes ferroptosis in sorafenib-induced cardiotoxicity by suppressing slc7a11 expression. Front Pharmacol. 2022;13: 904314. 36210815 10.3389/fphar.2022.904314 PMC9537618

[104] Jiang H, Wang C, Zhang A, Li Y, Li J, Li Z, et al. Atf4 protects against sorafenib-induced cardiotoxicity by suppressing ferroptosis. Biomed Pharmacother. 2022;153: 113280. 35724508 10.1016/j.biopha.2022.113280

[105] Li Y, Yan J, Sun H, Liang Y, Zhao Q, Yu S, et al. Ferroptosis inhibitor alleviates sorafenib-induced cardiotoxicity by attenuating KLF11-mediated FSP1-dependent ferroptosis. Int J Biol Sci. 2024;20:2622–39. 38725840 10.7150/ijbs.86479 PMC11077382

[106] León-Mateos L, Mosquera J, Antón AL. Treatment of sunitinib-induced hypertension in solid tumor by nitric oxide donors. Redox Biol. 2015;6:421–5. 26386874 10.1016/j.redox.2015.09.007 PMC4588456

[107] Kamba T, McDonald DM. Mechanisms of adverse effects of anti-VEGF therapy for cancer. Br J Cancer. 2007;96:1788–95. 17519900 10.1038/sj.bjc.6603813 PMC2359962

[108] Meyer T, Robles-Carrillo L, Robson T, Langer F, Desai H, Davila M, et al. Bevacizumab immune complexes activate platelets and induce thrombosis in FCGR2A transgenic mice. J Thromb Haemost. 2009;7:171–81. 18983497 10.1111/j.1538-7836.2008.03212.x

[109] Tzogani K, Skibeli V, Westgaard I, Dalhus M, Thoresen H, Slot KB, et al. The European Medicines Agency approval of axitinib (inlyta) for the treatment of advanced renal cell carcinoma after failure of prior treatment with sunitinib or a cytokine: summary of the scientific assessment of the committee for medicinal products for human use. Oncologist. 2015;20:196–201. 25616431 10.1634/theoncologist.2014-0177 PMC4319625

[110] Motzer RJ, Escudier B, Tomczak P, Hutson TE, Michaelson MD, Negrier S, et al. Axitinib versus sorafenib as second-line treatment for advanced renal cell carcinoma: overall survival analysis and updated results from a randomised phase 3 trial. Lancet Oncol. 2013;14:552–62. 23598172 10.1016/S1470-2045(13)70093-7

[111] Wilhelm SM, Dumas J, Adnane L, Lynch M, Carter CA, Schütz G, et al. Regorafenib (BAY 73-4506): a new oral multikinase inhibitor of angiogenic, stromal and oncogenic receptor tyrosine kinases with potent preclinical antitumor activity. Int J Cancer. 2011;129:245–55. 21170960 10.1002/ijc.25864

[112] Ettrich TJ, Seufferlein T. Regorafenib. Recent Results Cancer Res. 2018;211:45–56. 30069758 10.1007/978-3-319-91442-8_3

[113] Demetri GD, Reichardt P, Kang YK, Blay JY, Rutkowski P, Gelderblom H, et al. Efficacy and safety of regorafenib for advanced gastrointestinal stromal tumours after failure of imatinib and sunitinib (GRID): an international, multicentre, randomised, placebo-controlled, phase 3 trial. Lancet. 2013;381:295–302. 23177515 10.1016/S0140-6736(12)61857-1 PMC3819942

[114] Li J, Qin S, Xu R, Yau TC, Ma B, Pan H, et al. Regorafenib plus best supportive care versus placebo plus best supportive care in Asian patients with previously treated metastatic colorectal cancer (CONCUR): a randomised, double-blind, placebo-controlled, phase 3 trial. Lancet Oncol. 2015;16:619–29. 25981818 10.1016/S1470-2045(15)70156-7

[115] Commander H, Whiteside G, Perry C. Vandetanib: first global approval. Drugs. 2011;71:1355–65. 21770481 10.2165/11595310-000000000-00000

[116] Zang J, Wu S, Tang L, Xu X, Bai J, Ding C, et al. Incidence and risk of QTc interval prolongation among cancer patients treated with vandetanib: a systematic review and meta-analysis. PLoS ONE. 2012;7: e30353. 22363427 10.1371/journal.pone.0030353 PMC3281826

[117] Qi WX, Shen Z, Lin F, Sun YJ, Min DL, Tang LN, et al. Incidence and risk of hypertension with vandetanib in cancer patients: a systematic review and meta-analysis of clinical trials. Br J Clin Pharmacol. 2013;75:919–30. 22882307 10.1111/j.1365-2125.2012.04417.x PMC3612709

[118] Won E, Basunia A, Chatila WK, Hechtman JF, Chou JF, Ku GY, et al. Efficacy of combined VEGFR1-3, PDGFΑ/β, and FGFR1-3 blockade using nintedanib for esophagogastric cancer. Clin Cancer Res. 2019;25:3811–7. 30952642 10.1158/1078-0432.CCR-18-3789 PMC6606369

[119] Valade E, Dosne AG, Xie H, Kleiman R, Li LY, Perez-Ruixo JJ, et al. Assessment of the effect of erdafitinib on cardiac safety: analysis of ECGs and exposure-QTc in patients with advanced or refractory solid tumors. Cancer Chemother Pharmacol. 2019;84:621–33. 31280362 10.1007/s00280-019-03896-1

[120] Kim SY, Kim SM, Chang H, Kim BW, Lee YS, Chang HS, et al. Safety of tyrosine kinase inhibitors in patients with differentiated thyroid cancer: real-world use of lenvatinib and sorafenib in Korea. Front Endocrinol. 2019;10:384. 10.3389/fendo.2019.00384 PMC6581694 31244783

[121] Wirth LJ, Tahara M, Robinson B, Francis S, Brose MS, Habra MA, et al. Treatment-emergent hypertension and efficacy in the phase 3 study of (E7080) lenvatinib in differentiated cancer of the thyroid (SELECT). Cancer. 2018;124:2365–72. 29656442 10.1002/cncr.31344

[122] Pinkhas D, Ho T, Smith S. Assessment of pazopanib-related hypertension, cardiac dysfunction and identification of clinical risk factors for their development. Cardiooncology. 2017;3:1–4. 29497565 10.1186/s40959-017-0024-8 PMC5828231

[123] Li J, Qin S, Xu J, Xiong J, Wu C, Bai Y, et al. Randomized, double-blind, placebo-controlled phase III trial of apatinib in patients with chemotherapy-refractory advanced or metastatic adenocarcinoma of the stomach or gastroesophageal junction. J Clin Oncol. 2016;34:1448–54. 26884585 10.1200/JCO.2015.63.5995

[124] Wang W, He Q, Li C, Zhuang C, Zhang H, Wang Q, et al. Research on the mechanism and prevention of hypertension caused by apatinib through the RhoA/ROCK signaling pathway in a mouse model of gastric cancer. Front Cardiovasc Med. 2022;9: 873829. 35811723 10.3389/fcvm.2022.873829 PMC9262125

[125] Han B, Li K, Wang Q, Zhang L, Shi J, Wang Z, et al. Effect of anlotinib as a third-line or further treatment on overall survival of patients with advanced non-small cell lung cancer: the ALTER 0303 phase 3 randomized clinical trial. JAMA Oncol. 2018;4:1569–75. 30098152 10.1001/jamaoncol.2018.3039 PMC6248083

[126] Fruquintinib SM. First global approval. Drugs. 2018;78:1757–61. 30357594 10.1007/s40265-018-0998-z

[127] Dasari A, Lonardi S, Garcia-Carbonero R, Elez E, Yoshino T, Sobrero A, et al. Fruquintinib versus placebo in patients with refractory metastatic colorectal cancer (FRESCO-2): an international, multicentre, randomised, double-blind, phase 3 study. Lancet. 2023;402:41–53. 37331369 10.1016/S0140-6736(23)00772-9

[128] Xu J, Shen L, Bai C, Wang W, Li J, Yu X, et al. Surufatinib in advanced pancreatic neuroendocrine tumours (SANET-p): a randomised, double-blind, placebo-controlled, phase 3 study. Lancet Oncol. 2020;21:1489–99. 32966810 10.1016/S1470-2045(20)30493-9

[129] Jimenez-Fonseca P. Use of multikinase inhibitors/lenvatinib in patients with high cardiovascular risk/vasculopathy and radioiodine refractory-differentiated thyroid cancer. Cancer Med. 2022;11(Suppl 1):17–25. 36202605 10.1002/cam4.5127 PMC9537056

[130] Quintás-Cardama A, Cortes J. Molecular biology of bcr-abl1-positive chronic myeloid leukemia. Blood. 2009;113:1619–30. 18827185 10.1182/blood-2008-03-144790 PMC3952549

[131] Deininger M, Buchdunger E, Druker BJ. The development of imatinib as a therapeutic agent for chronic myeloid leukemia. Blood. 2005;105:2640–53. 15618470 10.1182/blood-2004-08-3097

[132] Distler JH, Distler O. Cardiotoxicity of imatinib mesylate: an extremely rare phenomenon or a major side effect? Ann Rheum Dis. 2007;66:836. 17513571 10.1136/ard.2006.067710 PMC1954675

[133] Atallah E, Durand JB, Kantarjian H, Cortes J. Congestive heart failure is a rare event in patients receiving imatinib therapy. Blood. 2007;110:1233–7. 17449798 10.1182/blood-2007-01-070144

[134] Trent JC, Patel SS, Zhang J, Araujo DM, Plana JC, Lenihan DJ, et al. Rare incidence of congestive heart failure in gastrointestinal stromal tumor and other sarcoma patients receiving imatinib mesylate. Cancer. 2010;116:184–92. 19885836 10.1002/cncr.24683 PMC4306337

[135] Kerkelä R, Grazette L, Yacobi R, Iliescu C, Patten R, Beahm C, et al. Cardiotoxicity of the cancer therapeutic agent imatinib mesylate. Nat Med. 2006;12:908–16. 16862153 10.1038/nm1446

[136] Steinberg SF. Distinctive activation mechanisms and functions for protein kinase cdelta. Biochem J. 2004;384:449–59. 15491280 10.1042/BJ20040704 PMC1134130

[137] Park YH, Park HJ, Kim BS, Ha E, Jung KH, Yoon SH, et al. BNP as a marker of the heart failure in the treatment of imatinib mesylate. Cancer Lett. 2006;243:16–22. 16388897 10.1016/j.canlet.2005.11.014

[138] Maharsy W, Aries A, Mansour O, Komati H, Nemer M. Ageing is a risk factor in imatinib mesylate cardiotoxicity. Eur J Heart Fail. 2014;16:367–76. 24504921 10.1002/ejhf.58 PMC4238824

[139] Marslin G, Revina AM, Khandelwal VK, Balakumar K, Prakash J, Franklin G, et al. Delivery as nanoparticles reduces imatinib mesylate-induced cardiotoxicity and improves anticancer activity. Int J Nanomed. 2015;10:3163–70. 10.2147/IJN.S75962 PMC4425327 25995626

[140] Fernández A, Sanguino A, Peng Z, Ozturk E, Chen J, Crespo A, et al. An anticancer C-kit kinase inhibitor is reengineered to make it more active and less cardiotoxic. J Clin Invest. 2007;117:4044–54. 18060038 10.1172/JCI32373 PMC2104494

[141] Sarszegi Z, Bognar E, Gaszner B, Kónyi A, Gallyas F Jr, Sumegi B, et al. BGP-15, a PARP-inhibitor, prevents imatinib-induced cardiotoxicity by activating akt and suppressing JNK and p38 MAP kinases. Mol Cell Biochem. 2012;365:129–37. 22350755 10.1007/s11010-012-1252-8

[142] National comprehensive cancer network: chronic myeloid leukemia (version 3.2022). 2022.

[143] Shah NP, Tran C, Lee FY, Chen P, Norris D, Sawyers CL. Overriding imatinib resistance with a novel ABL kinase inhibitor. Science. 2004;305:399–401. 15256671 10.1126/science.1099480

[144] Lindauer M, Hochhaus A. Dasatinib. Recent Results Cancer Res. 2018;212:29–68. 30069624 10.1007/978-3-319-91439-8_2

[145] Dahlén T, Edgren G, Ljungman P, Flygt H, Richter J, Olsson-Strömberg U, et al. Adverse outcomes in chronic myeloid leukemia patients treated with tyrosine kinase inhibitors: follow-up of patients diagnosed 2002–2017 in a complete coverage and nationwide agnostic register study. Am J Hematol. 2022;97:421–30. 35015312 10.1002/ajh.26463 PMC9306877

[146] Guignabert C, Phan C, Seferian A, Huertas A, Tu L, Thuillet R, et al. Dasatinib induces lung vascular toxicity and predisposes to pulmonary hypertension. J Clin Invest. 2016;126:3207–18. 27482885 10.1172/JCI86249 PMC5004960

[147] Cortes JE, Kim DW, Pinilla-Ibarz J, le Coutre PD, Paquette R, Chuah C, et al. Ponatinib efficacy and safety in Philadelphia chromosome-positive leukemia: final 5-year results of the phase 2 PACE trial. Blood. 2018;132:393–404. 29567798 10.1182/blood-2016-09-739086 PMC6071555

[148] Jiang Q, Li Z, Qin Y, Li W, Xu N, Liu B, et al. Olverembatinib (HQP1351), a well-tolerated and effective tyrosine kinase inhibitor for patients with T315I-mutated chronic myeloid leukemia: results of an open-label, multicenter phase 1/2 trial. J Hematol Oncol. 2022;15:113. 35982483 10.1186/s13045-022-01334-z PMC9389804

[149] Tousif S, Singh AP, Umbarkar P, Galindo C, Wheeler N, Toro CA, et al. Ponatinib drives cardiotoxicity by S100A8/A9-NLRP3-IL-1β mediated inflammation. Circ Res. 2023;132:267–89. 36625265 10.1161/CIRCRESAHA.122.321504 PMC9898181

[150] Singh AP, Glennon MS, Umbarkar P, Gupte M, Galindo CL, Zhang Q, et al. Ponatinib-induced cardiotoxicity: delineating the signalling mechanisms and potential rescue strategies. Cardiovasc Res. 2019;115:966–77. 30629146 10.1093/cvr/cvz006 PMC6452321

[151] Hnatiuk AP, Bruyneel AAN, Tailor D, Pandrala M, Dheeraj A, Li W, et al. Reengineering ponatinib to minimize cardiovascular toxicity. Cancer Res. 2022;82:2777–91. 35763671 10.1158/0008-5472.CAN-21-3652 PMC9620869

[152] Pandrala M, Bruyneel AAN, Hnatiuk AP, Mercola M, Malhotra SV. Designing novel BCR-ABL inhibitors for chronic myeloid leukemia with improved cardiac safety. J Med Chem. 2022;65:10898–919. 35944901 10.1021/acs.jmedchem.1c01853 PMC9421657

[153] Yu R, Ai N, Huang C, Wang D, Bian C, Ge W, et al. Aspirin reduces ponatinib-induced cardiovascular toxic phenotypes and death in zebrafish. Biomed Pharmacother. 2024;180: 117503. 39357328 10.1016/j.biopha.2024.117503

[154] Yu B, Osman AEG, Sladojevic N, Prabhu N, Tai HC, Chen D, et al. Involvement of rho-associated coiled-coil containing kinase (ROCK) in BCR-ABL1 tyrosine kinase inhibitor cardiovascular toxicity. JACC CardioOncol. 2022;4:371–83. 36213346 10.1016/j.jaccao.2022.06.004 PMC9537085

[155] Zain R, Vihinen M. Structure-function relationships of covalent and non-covalent BTK inhibitors. Front Immunol. 2021;12: 694853. 34349760 10.3389/fimmu.2021.694853 PMC8328433

[156] de Claro RA, McGinn KM, Verdun N, Lee SL, Chiu HJ, Saber H, et al. Fda approval: ibrutinib for patients with previously treated mantle cell lymphoma and previously treated chronic lymphocytic leukemia. Clin Cancer Res. 2015;21:3586–90. 26275952 10.1158/1078-0432.CCR-14-2225

[157] Kim MS, Prasad V. Us Food and Drug Administration approvals for Bruton tyrosine kinase inhibitors in patients with chronic lymphocytic leukemia: potential inefficiencies in trial design and evidence generation. Cancer. 2020;126:4270–2. 32644193 10.1002/cncr.33058

[158] Buck B, Chum AP, Patel M, Carter R, Nawaz H, Yildiz V, et al. Cardiovascular magnetic resonance imaging in patients with ibrutinib-associated cardiotoxicity. JAMA Oncol. 2023;9:552–5. 36729480 10.1001/jamaoncol.2022.6869 PMC9896369

[159] Abdel-Qadir H, Sabrie N, Leong D, Pang A, Austin PC, Prica A, et al. Cardiovascular risk associated with ibrutinib use in chronic lymphocytic leukemia: a population-based cohort study. J Clin Oncol. 2021;39:3453–62. 34464154 10.1200/JCO.21.00693

[160] Update on imbruvica® (ibrutinib) U.S. Accelerated approvals for mantle cell lymphoma and marginal zone lymphoma indications. News release. Johnson & Johnson and Pharmacyclics. 2023. https://bit.ly/3ZUpDnJ. Accessed 10 Apr 2023.

[161] Leong DP, Caron F, Hillis C, Duan A, Healey JS, Fraser G, et al. The risk of atrial fibrillation with ibrutinib use: a systematic review and meta-analysis. Blood. 2016;128:138–40. 27247135 10.1182/blood-2016-05-712828

[162] Khountham S, Shindiapina P, Mo X, Lachowiez C, Wiczer T, Mousa L, et al. Natural history of noninfectious, ibrutinib-attributable adverse events in patients with chronic lymphocytic leukemia. Leuk Lymphoma. 2021;62:716–21. 33210562 10.1080/10428194.2020.1838508

[163] Xiao L, Salem JE, Clauss S, Hanley A, Bapat A, Hulsmans M, et al. Ibrutinib-mediated atrial fibrillation attributable to inhibition of c-terminal src kinase. Circulation. 2020;142:2443–55. 33092403 10.1161/CIRCULATIONAHA.120.049210 PMC9661397

[164] Jiang L, Li L, Ruan Y, Zuo S, Wu X, Zhao Q, et al. Ibrutinib promotes atrial fibrillation by inducing structural remodeling and calcium dysregulation in the atrium. Heart Rhythm. 2019;16:1374–82. 30959203 10.1016/j.hrthm.2019.04.008

[165] Pretorius L, Du XJ, Woodcock EA, Kiriazis H, Lin RC, Marasco S, et al. Reduced phosphoinositide 3-kinase (p110alpha) activation increases the susceptibility to atrial fibrillation. Am J Pathol. 2009;175:998–1009. 19679877 10.2353/ajpath.2009.090126 PMC2731119

[166] McMullen JR, Boey EJ, Ooi JY, Seymour JF, Keating MJ, Tam CS. Ibrutinib increases the risk of atrial fibrillation, potentially through inhibition of cardiac PI3K-Akt signaling. Blood. 2014;124:3829–30. 25498454 10.1182/blood-2014-10-604272

[167] Guha A, Derbala MH, Zhao Q, Wiczer TE, Woyach JA, Byrd JC, et al. Ventricular arrhythmias following ibrutinib initiation for lymphoid malignancies. J Am Coll Cardiol. 2018;72:697–8. 30072003 10.1016/j.jacc.2018.06.002 PMC7529121

[168] Ratain MJ, Moslehi JJ, Lichter AS. Ibrutinib’s cardiotoxicity-an opportunity for postmarketing regulation. JAMA Oncol. 2021;7:177–8. 33237281 10.1001/jamaoncol.2020.5742

[169] Lampson BL, Yu L, Glynn RJ, Barrientos JC, Jacobsen ED, Banerji V, et al. Ventricular arrhythmias and sudden death in patients taking ibrutinib. Blood. 2017;129:2581–4. 28223277 10.1182/blood-2016-10-742437 PMC7219062

[170] Pan Y, Zhao Y, Ren H, Wang X, Liu C, Du B, et al. Epidemiology, clinical characteristics and potential mechanism of ibrutinib-induced ventricular arrhythmias. Front Pharmacol. 2024;15:1513913. 39629084 10.3389/fphar.2024.1513913 PMC11611568

[171] Salem JE, Manouchehri A, Bretagne M, Lebrun-Vignes B, Groarke JD, Johnson DB, et al. Cardiovascular toxicities associated with ibrutinib. J Am Coll Cardiol. 2019;74:1667–78. 31558250 10.1016/j.jacc.2019.07.056

[172] Kudinov A, Darbar D. Deciphering the electrophysiological mechanisms for ibrutinib-induced ventricular arrhythmias. JACC CardioOncol. 2020;2:630–1. 34396274 10.1016/j.jaccao.2020.10.001 PMC8352279

[173] Morissette MP, Susser SE, Stammers AN, Moffatt TL, Wigle JT, Wigle TJ, et al. Exercise-induced increases in the expression and activity of cardiac sarcoplasmic reticulum calcium ATPase 2 is attenuated in AMPKα(2) kinase-dead mice. Can J Physiol Pharmacol. 2019;97:786–95. 31237455 10.1139/cjpp-2018-0737

[174] Zhao Y, Du B, Chakraborty P, Denham N, Massé S, Lai PFH, et al. Impaired cardiac AMPK (5′-adenosine monophosphate-activated protein kinase) and ca(2+)-handling, and action potential duration heterogeneity in ibrutinib-induced ventricular arrhythmia vulnerability. J Am Heart Assoc. 2024;13: e032357. 38842296 10.1161/JAHA.123.032357 PMC11255774

[175] Li P, Liu D, Gao P, Yuan M, Zhao Z, Zhang Y, et al. Mitigating ibrutinib-induced ventricular arrhythmia and cardiac dysfunction with metformin. Cancer Innov. 2025;4: e151. 39544722 10.1002/cai2.151 PMC11560382

[176] Du B, Chakraborty P, Azam MA, Massé S, Lai PFH, Niri A, et al. Acute effects of ibrutinib on ventricular arrhythmia in spontaneously hypertensive rats. JACC CardioOncol. 2020;2:614–29. 34396273 10.1016/j.jaccao.2020.08.012 PMC8352013

[177] Brown JR, Moslehi J, O’Brien S, Ghia P, Hillmen P, Cymbalista F, et al. Characterization of atrial fibrillation adverse events reported in ibrutinib randomized controlled registration trials. Haematologica. 2017;102:1796–805. 28751558 10.3324/haematol.2017.171041 PMC5622864

[178] Jonas DE, Kahwati LC, Yun JDY, Middleton JC, Coker-Schwimmer M, Asher GN. Screening for atrial fibrillation with electrocardiography: evidence report and systematic review for the us preventive services task force. JAMA. 2018;320:485–98. 30088015 10.1001/jama.2018.4190

[179] Markham A, Dhillon S. Acalabrutinib: first global approval. Drugs. 2018;78:139–45. 29209955 10.1007/s40265-017-0852-8

[180] Danilov AV, Persky DO. Incorporating acalabrutinib, a selective next-generation bruton tyrosine kinase inhibitor, into clinical practice for the treatment of haematological malignancies. Br J Haematol. 2021;193:15–25. 33216986 10.1111/bjh.17184

[181] Bhat SA, Gambril J, Azali L, Chen ST, Rosen L, Palettas M, et al. Ventricular arrhythmias and sudden death events following acalabrutinib initiation. Blood. 2022;140:2142–5. 35917449 10.1182/blood.2022016953 PMC10405526

[182] Furman RR, Byrd JC, Owen RG, O’Brien SM, Brown JR, Hillmen P, et al. Pooled analysis of safety data from clinical trials evaluating acalabrutinib monotherapy in mature B-cell malignancies. Leukemia. 2021;35:3201–11. 33907299 10.1038/s41375-021-01252-y

[183] Brown JR, Byrd JC, Ghia P, Sharman JP, Hillmen P, Stephens DM, et al. Pooled analysis of cardiovascular events from clinical trials evaluating acalabrutinib monotherapy in patients with chronic lymphocytic leukemia (CLL). Blood. 2020;136:52–4.

[184] Brown JR, Byrd JC, Ghia P, Sharman JP, Hillmen P, Stephens DM, et al. Cardiovascular adverse events in patients with chronic lymphocytic leukemia receiving acalabrutinib monotherapy: pooled analysis of 762 patients. Haematologica. 2022;107:1335–46. 34587719 10.3324/haematol.2021.278901 PMC9152976

[185] Byrd JC, Hillmen P, Ghia P, Kater AP, Chanan-Khan A, Furman RR, et al. Acalabrutinib versus ibrutinib in previously treated chronic lymphocytic leukemia: results of the first randomized phase III trial. J Clin Oncol. 2021;39:3441–52. 34310172 10.1200/JCO.21.01210 PMC8547923

[186] Seymour JF, Byrd JC, Ghia P, Kater AP, Chanan-Khan AA, Furman RR, et al. Detailed safety profile of acalabrutinib vs ibrutinib in previously treated chronic lymphocytic leukemia in elevate-rr. Blood. 2023. 10.1182/blood.2022018818. 37390310 10.1182/blood.2022018818 PMC10644206

[187] Awan FT, Schuh A, Brown JR, Furman RR, Pagel JM, Hillmen P, et al. Acalabrutinib monotherapy in patients with chronic lymphocytic leukemia who are intolerant to ibrutinib. Blood Adv. 2019;3:1553–62. 31088809 10.1182/bloodadvances.2018030007 PMC6517672

[188] Syed YY. Zanubrutinib: first approval. Drugs. 2020;80:91–7. 31933167 10.1007/s40265-019-01252-4

[189] Hillmen P, Eichhorst B, Brown JR, Lamanna N, O’Brien SM, Tam CS, et al. Zanubrutinib versus ibrutinib in relapsed/refractory chronic lymphocytic leukemia and small lymphocytic lymphoma: Interim analysis of a randomized phase III trial. J Clin Oncol. 2023;41:1035–45. 36395435 10.1200/JCO.22.00510 PMC9928683

[190] Lee DH, Hawk F, Seok K, Gliksman M, Emole J, Rhea IB, et al. Association between ibrutinib treatment and hypertension. Heart. 2022;108:445–50. 34210750 10.1136/heartjnl-2021-319110 PMC9809112

[191] Burger JA, Barr PM, Robak T, Owen C, Ghia P, Tedeschi A, et al. Long-term efficacy and safety of first-line ibrutinib treatment for patients with CLL/SLL: 5 years of follow-up from the phase 3 RESONATE-2 study. Leukemia. 2020;34:787–98. 31628428 10.1038/s41375-019-0602-x PMC7214263

[192] Byrd JC, Furman RR, Coutre SE, Flinn IW, Burger JA, Blum K, et al. Ibrutinib treatment for first-line and relapsed/refractory chronic lymphocytic leukemia: final analysis of the pivotal phase ib/II PCYC-1102 study. Clin Cancer Res. 2020;26:3918–27. 32209572 10.1158/1078-0432.CCR-19-2856 PMC8175012

[193] Dickerson T, Wiczer T, Waller A, Philippon J, Porter K, Haddad D, et al. Hypertension and incident cardiovascular events following ibrutinib initiation. Blood. 2019;134:1919–28. 31582362 10.1182/blood.2019000840 PMC6887116

[194] Chen ST, Azali L, Rosen L, Zhao Q, Wiczer T, Palettas M, et al. Hypertension and incident cardiovascular events after next-generation BTKi therapy initiation. J Hematol Oncol. 2022;15:92. 35836241 10.1186/s13045-022-01302-7 PMC9281099

[195] Dimopoulos MA, Opat S, D’Sa S, Jurczak W, Lee HP, Cull G, et al. Zanubrutinib versus ibrutinib in symptomatic Waldenström macroglobulinemia: final analysis from the randomized phase III ASPEN study. J Clin Oncol. 2023;41:5099–106. 37478390 10.1200/JCO.22.02830 PMC10666987

[196] Tam CS, Dimopoulos M, Garcia-Sanz R, Trotman J, Opat S, Roberts AW, et al. Pooled safety analysis of zanubrutinib monotherapy in patients with B-cell malignancies. Blood Adv. 2022;6:1296–308. 34724705 10.1182/bloodadvances.2021005621 PMC8864647

[197] Awan FT, Addison D, Alfraih F, Baratta SJ, Campos RN, Cugliari MS, et al. International consensus statement on the management of cardiovascular risk of bruton’s tyrosine kinase inhibitors in cll. Blood Adv. 2022;6:5516–25. 35790105 10.1182/bloodadvances.2022007938 PMC9631706

[198] Ekram J, Rathore A, Avila C, Hussein R, Alomar M. Unveiling the cardiotoxicity conundrum: navigating the seas of tyrosine kinase inhibitor therapies. Cancer Control. 2024;31:10732748241285756. 39318033 10.1177/10732748241285755 PMC11440564

[199] Brown TM, Pack QR, Aberegg E, Brewer LC, Ford YR, Forman DE, et al. Core components of cardiac rehabilitation programs: 2024 update: a scientific statement from the american heart association and the american association of cardiovascular and pulmonary rehabilitation. Circulation. 2024;150:e328–47. 39315436 10.1161/CIR.0000000000001289

[200] Van Horn L, Carson JA, Appel LJ, Burke LE, Economos C, Karmally W, et al. Recommended dietary pattern to achieve adherence to the American Heart Association/American College of Cardiology (AHA/ACC) guidelines: a scientific statement from the american heart association. Circulation. 2016;134:e505–29. 27789558 10.1161/CIR.0000000000000462

[201] Gilchrist SC, Barac A, Ades PA, Alfano CM, Franklin BA, Jones LW, et al. Cardio-oncology rehabilitation to manage cardiovascular outcomes in cancer patients and survivors: a scientific statement from the American Heart Association. Circulation. 2019;139:e997–1012. 30955352 10.1161/CIR.0000000000000679 PMC7603804

[202] Sun W, Zhang H, Guo J, Zhang X, Zhang L, Li C, et al. Comparison of the efficacy and safety of different ACE inhibitors in patients with chronic heart failure: a PRISMA-compliant network meta-analysis. Medicine. 2016;95: e2554. 26871774 10.1097/MD.0000000000002554 PMC4753869

[203] Lyon AR, López-Fernández T, Couch LS, Asteggiano R, Aznar MC, Bergler-Klein J, et al. 2022 esc guidelines on cardio-oncology developed in collaboration with the European Hematology Association (EHA), the European Society for Therapeutic Radiology and Oncology (ESTRO) and the International Cardio-Oncology Society (IC-OS). Eur Heart J. 2022;43:4229–361. 36017568 10.1093/eurheartj/ehac244

